# A draft *Diabrotica virgifera virgifera* genome: insights into control and host plant adaption by a major maize pest insect

**DOI:** 10.1186/s12864-022-08990-y

**Published:** 2023-01-13

**Authors:** Brad S. Coates, Kimberly K. O. Walden, Dimpal Lata, Neetha Nanoth Vellichirammal, Robert F. Mitchell, Martin N. Andersson, Rachel McKay, Marcé D. Lorenzen, Nathaniel Grubbs, Yu-Hui Wang, Jinlong Han, Jing Li Xuan, Peter Willadsen, Huichun Wang, B. Wade French, Raman Bansal, Sammy Sedky, Dariane Souza, Dakota Bunn, Lance J. Meinke, Nicholas J. Miller, Blair D. Siegfried, Thomas W. Sappington, Hugh M. Robertson

**Affiliations:** 1 Corn Insects & Crop Genetics Research Unit, USDA-ARS, 2310 Pammel Dr, 532 Science II, Iowa State University, Ames, IA 50011 USA; 2 Roy J. Carver Biotechnology Center, University of Illinois at Champaign-Urbana, Urbana, IL USA; 3 Department of Biology, Illinois Institute of Technology, Chicago, IL USA; 4 University of Nebraska Medical Center, Omaha, NE USA; 5 University of Wisconsin Oshkosh, Oshkosh, WI USA; 6 Department of Biology, Lund University, Lund, Sweden; 7 Department of Entomology and Plant Pathology, North Carolina State University, Raleigh, NC USA; 8 Department of Entomology, University of Nebraska, Lincoln, NE USA; 9 Integrated Crop Systems Research Unit, USDA-ARS, Brookings, SD USA; 10 USDA-ARS, San Joaquin Valley Agricultural Sciences Center, Parlier, CA USA; 11 Department of Entomology, University of Florida, Gainesville, FL USA; 12 Department of Entomology, University of Illinois at Champaign-Urbana, Urbana, IL USA

**Keywords:** Genome assembly, Host plant specialization, Differential expression

## Abstract

**Background:**

Adaptations by arthropod pests to host plant defenses of crops determine their impacts on agricultural production. The larval host range of western corn rootworm, *Diabrotica virgifera virgifera* (Coleoptera: Chrysomelidae), is restricted to maize and a few grasses. Resistance of *D. v. virgifera* to crop rotation practices and multiple insecticides contributes to its status as the most damaging pest of cultivated maize in North America and Europe. The extent to which adaptations by this pest contributes to host plant specialization remains unknown.

**Results:**

A 2.42 Gb draft *D. v. virgifera* genome, Dvir_v2.0, was assembled from short shotgun reads and scaffolded using long-insert mate-pair, transcriptome and linked read data. K-mer analysis predicted a repeat content of ≥ 61.5%. Ortholog assignments for Dvir_2.0 RefSeq models predict a greater number of species-specific gene duplications, including expansions in ATP binding cassette transporter and chemosensory gene families, than in other Coleoptera. A majority of annotated *D. v. virgifera* cytochrome P450s belong to CYP4, 6, and 9 clades. A total of 5,404 transcripts were differentially-expressed between *D. v. virgifera* larvae fed maize roots compared to alternative host (*Miscanthus*), a marginal host (*Panicum virgatum*), a poor host (*Sorghum bicolor*) and starvation treatments; Among differentially-expressed transcripts, 1,908 were shared across treatments and the least number were between *Miscanthus* compared to maize. Differentially-expressed transcripts were enriched for putative spliceosome, proteosome, and intracellular transport functions. General stress pathway functions were unique and enriched among up-regulated transcripts in marginal host, poor host, and starvation responses compared to responses on primary (maize) and alternate hosts.

**Conclusions:**

Manual annotation of *D. v. virgifera* Dvir_2.0 RefSeq models predicted expansion of paralogs with gene families putatively involved in insecticide resistance and chemosensory perception. Our study also suggests that adaptations of *D. v. virgifera* larvae to feeding on an alternate host plant invoke fewer transcriptional changes compared to marginal or poor hosts. The shared up-regulation of stress response pathways between marginal host and poor host, and starvation treatments may reflect nutrient deprivation. This study provides insight into transcriptomic responses of larval feeding on different host plants and resources for genomic research on this economically significant pest of maize.

**Supplementary Information:**

The online version contains supplementary material available at 10.1186/s12864-022-08990-y.

## Introduction

Herbivorous pest insects adversely impact the production of food, fiber and biofuel crops. The range of agricultural host plants an insect species infests is influenced by the evolutionary “arms race” between plant defensive adaptations and insect countermeasures [1, 2], and involves complex interactions between physiological, biochemical, ecological, and behavioral factors [3–5]. These include female oviposition preferences (often involving chemosensory attraction) and accompanying physiological changes that facilitate evasion, detoxification, or sequestration of host plant defenses [6]. Female oviposition preference and subsequent performance of immature stages on a range of host plants are assumed to be co-adapted traits [7–10], as are optimized adult feeding and oviposition preferences [11]. Other factors such as predator and host abundances also impact host preference and performance [11, 12]. Oviposition host selection is not always congruent with progeny performance [13], and oviposition may occur in locations or under conditions in which non-optimal hosts are dominant in the landscape where a generalist insect herbivore resides [10]. In contrast, specialists will inevitably oviposit on a poor-quality host during times when resource limitation is geographically widespread [14]. Complex mechanisms are involved in host adaptations, in which perception and selection of hosts by chemosensory pathways are important [15], involving receptors and proteins encoded by several gene families, including odorant receptors (ORs), gustatory receptors (GRs), ionotropic receptors (IRs), and odorant binding proteins (OBPs).

The western corn rootworm, *Diabrotica virgifera virgifera* (LeConte) (Coleoptera: Chrysomelidae), is native to the high plains of North America. Populations are now established throughout the Midwest and northeastern United States and southern Ontario, Canada following an eastward range expansion which coincided with intensification of continuous maize production involving use of synthetic insecticides and fertilizers [16–18]. Establishment of this species in Europe occurred via multiple trans-Atlantic introductions since the 1980s and subsequent intra-continental spread [19, 20]. Populations are univoltine and overwinter as diapausing eggs in the soil. Eggs hatch the following spring, and subterranean larvae feed on and bore into host roots [21]. The *D. v. virgifera* host range is primarily limited to maize, although and a few wild grasses can support a low population level in the absence of maize [22–25]. Adult oviposition and larval feeding are documented on the bioenergy crops, *Miscanthus* (*Miscanthus* × *giganteus*) and switchgrass (*Panicum virgatum*), but frequencies are many-fold lower compared to maize [26]. Although oviposition occurs in some grass habitats, subsequent larval development is reduced compared to maize [27]. Furthermore, adult *D. v. virgifera* that feed on maize have greater longevity compared to other host plants, but maize volatiles appear to influence the oviposition in nearby non-maize hosts, despite preference for maize [28].

Larval feeding on roots of the preferred host, cultivated maize, causes reduced plant health and grain yield [29]. Most adults reside in or near natal fields, but can disperse long distances [30–34]. Beetles feed on maize silks (stamens) and are vectors of several plant viruses and pathogens [35, 36]. In-soil or foliar chemical insecticide applications historically were effective at protecting crop yield, but *D. v. virgifera* resistance is reducing efficacies of cyclodiene, organophosphate, carbamate, and pyrethroid classes [37]. Populations of *D. v. virgifera* have also evolved resistance to *Bacillus thuringiensis* (Bt) pesticidal proteins expressed by transgenic maize hybrids [38, 39]. Additionally, populations resistant to crop rotation evolved in east-central Illinois and the trait spread to several adjacent states [40]. Adults in rotation resistant populations not only oviposit in maize fields, but in other crops, most often soybean, *Glycine max*, the main rotation partner of maize in the Midwest United States, thus circumventing the efficacy of maize and *G. max* crop rotations for controlling this pest [16, 40–44]. Although rotation resistance ultimately reflects relaxed ovipositional fidelity to maize [44–46], the genetic and mechanistic basis remains unclear. Rotation resistance is associated with constitutive up-regulation of a cathepsin-L gene in the adult gut in response to *G. max* protease inhibitors [47] as well as changes in the gut microbiota [48], differences in responses to maize phenology [49, 50] and increased movement behavior [51–53]. 

Sustainable pest management strategies require an understanding of mechanisms by which a insects adapt to host plant defenses [54] since these adaptations may also contribute to the development of insecticide resistance [55–57]. Differences in larval performance and host range are associated with changes in expression of a suite of transcripts encoding detoxification enzymes and proteases [58–60], including cytochrome P450 monooxygenases and ATP binding cassette (ABC) transporters. Prior studies document the association of differentially-expressed *D. v. virgifera* transcripts with larval [61–63] or exposures [64] to Bt, resistance to chemical insecticides [65, 66], and between adults feeding on maize or *G. max* [67]. However, investigations into larval *D. v. virgifera* adaptations to maize and non-maize grass hosts have yet to be conducted. To facilitate research into the molecular basis of ecological and environmental adaptations, a draft whole genome sequence was assembled from short-read libraries for the *D. v. virgifera* inbred line, Ped12-6. Furthermore, prediction of differentially-expressed gene models between *D. v. virgifera* larvae exposed to maize compared to alternate, marginal, and poor hosts grasses demonstrate the plasticity in larval responses, and high degree of shared response to maize and alternate *Miscanthus* hosts. The genomic and data resources from this study are important for understanding adaptive responses associated with host plant range, as well as future evolutionary, ecological, and population genomic research in *D. v. virgifera* relevant to control of this problematic crop pest insect [68].

## Results

### Genome sequencing and scaffold assembly

A total of 1,650 million short paired-end (PE) and 584 million mate-pair (MP) reads were generated among libraries constructed from adult *D. v. virgifera* of the inbred line Ped12-6 (isolate Ped12-6-A-3; BioSample SAMN08631342) by sequencing across 15 Illumina HiSeq2000 lanes. This consisted of 297.7 and 116.9 Gb of PE and MP sequence data, respectively (table S1). Reads from ten PE ~ 500 bp insert libraries provided ~ 116.3-fold coverage of the 2.56 Gb flow cytometry estimated haploid (1 N) genome [69]. A maximum 1 N genome size of 2.60 Gb was estimated from a histogram of 31 nt k-mer occurrences in the ~ 500 bp PE read data (Fig S1; maximum 1.60 Gb and 1.00 Gb repeat and unique sequence lengths, respectively). The greatest number of unique k-mers occurred at a coverage of 24 and representative of the homozygous genome proportion (99.56%), whereas the k-mer peak at 12 is indicative of the heterozygous portion (0.46%). Estimated level of duplication was 0.331.

**Fig. 1:**
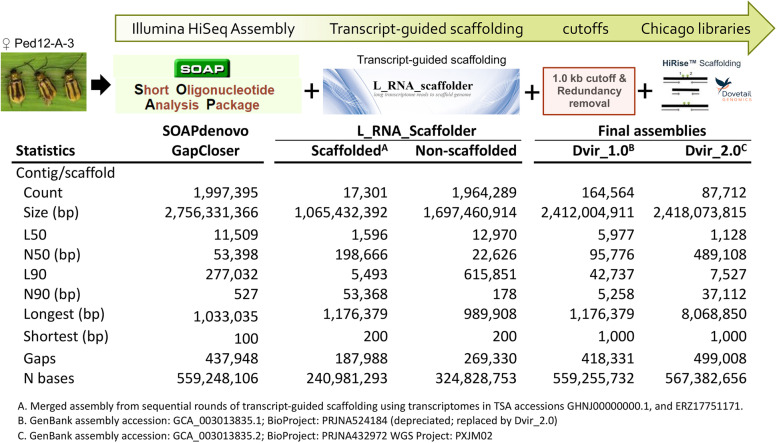
Approach and statistics during sequential assembly of the draft *Diabrotica virgifera virgifera* genome

Contigs were assembled from the ~ 500 bp insert shotgun library reads, followed by scaffolding via progressive incorporation of reads from larger PE and MP libraires and a final gap closure step. This resulted in a highly fragmented draft assembly of nearly 2 million contigs summing to 2.76 Gb (Fig. 1) with 0.56 Gb N content (gaps) and a contig N50 of 53.40 kb. To improve contiguity of the draft assembly, 47,284 contigs from the SOAPdenovo GapCloser assembly were placed into 13,725 scaffolds based on transcript information from two *D. v. virgifera* transcriptome resources used by L-RNA-Scaffolder (Fig. 1). Specifically, the round 1 transcript-guided assembly by L-RNA-Scaffolder resulted in the joining of 23,818 SOAPdenovo contigs into 8,683 scaffolds (list of joins in table S2). The subsequent round 2 by L-RNA-Scaffolder resulted in joining an additional 23,466 contigs from the unassembled portion of round 1 into 8,618 scaffolds (list of joins in table S3). This combined set of 17,301 transcript-guided scaffolds totaled 1.07 Gb with an N50 of 0.199 Mb (Fig. 1). The total number of Ns and gaps in the L_RNA_Scaffolder assembly increased by 6.56 Mb and 19,370, respectively, compared to the SOAPdenovo GapCloser assembly. This increase occurred due to L_RNA_Scaffolder insertion an estimated number of Ns between connections to represent putative intron spacing from calculated media intron size [70]. Redundant contigs and scaffolds were removed, as well as contigs < 1.0 kb, and led to a highly fragmented intermediate Dvir_1.0 assembly which was submitted to NCBI (Genome accession: GCA_003013835.1; depreciated) (Fig. 1; table S4). Predicted orthologs from the arthropoda_odb10 set (*n* = 1,013 orthologs) in Dvir_1.0 showed that 934 (92.2%) were complete, of which 90.5% were single copy. Fragmented and missing orthologs accounted for 5.9 and 1.9% of those in arthropoda_odb10, respectively.

**Fig. 2:**
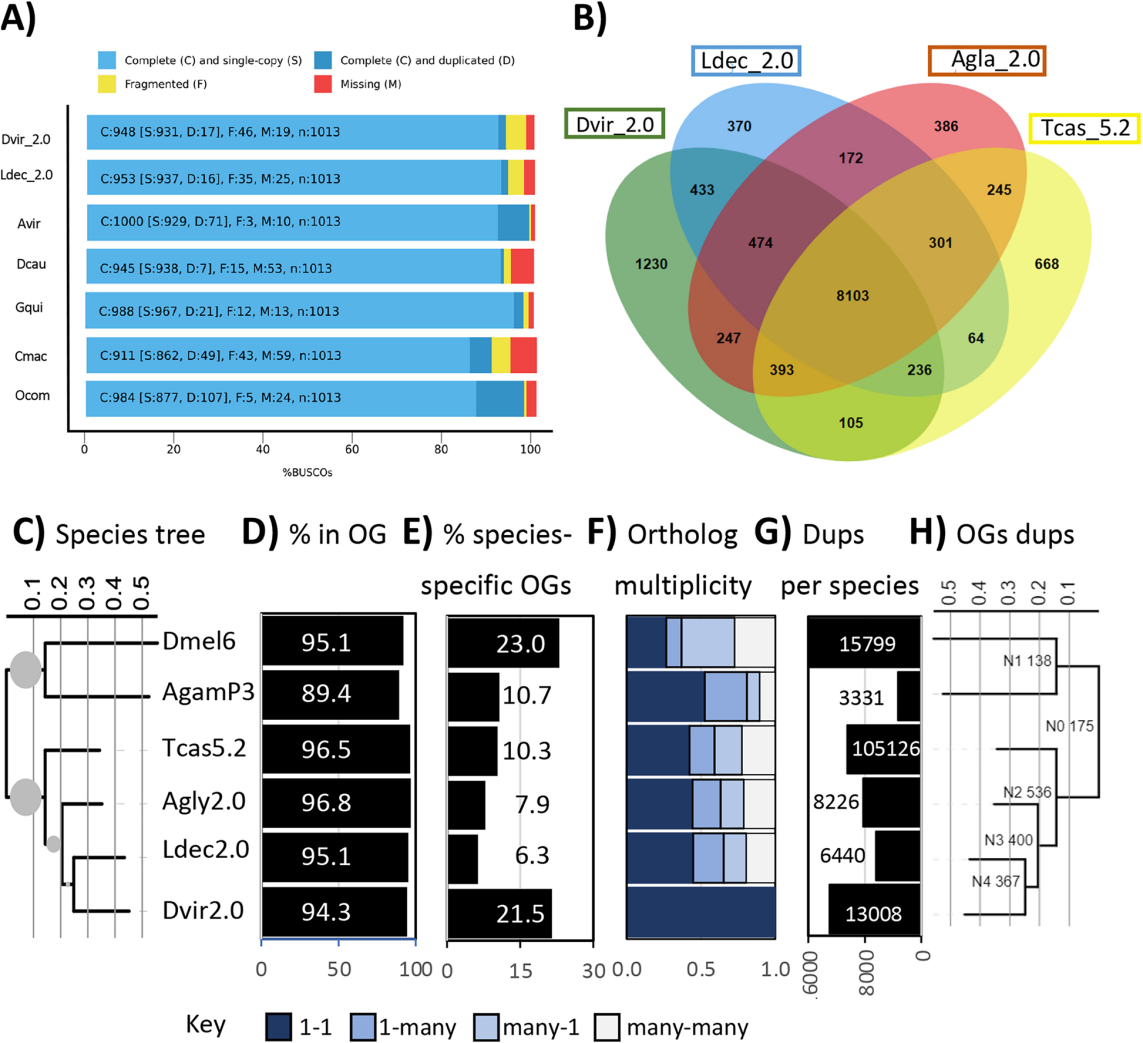
Comparative gene representation and content. **A** Comparison of Benchmarking Universal Single-Copy Orthologs (BUSCO) scores for 1013 Arthropoda_odb10 orthologs in the *D. v. virgifera* genome assembly, Dvir_2.0, with other representative assemblies from species in the Order Coleoptera, Family Chrysomelidae. **B** Overlap among ortholog groups (OGs) assigned to RefSeq protein models from *Diabrotica virgifera virgifera* (Dvir_2.0), *Leptinotarsa decemlineata* (Ldec_2.0), *Tribolium castaneum* (Tcas5.2), *Anopheles gambiae* (AgamP3), and *Drosophila melanogaster* (Dmel6). ***C*** Species tree constructed from RefSeq protein models with *Caenorhabditis elegans* (WBcel235) as outgroup. Scaled branch lengths shown and bootstrap node support indicated by circle size. **D** Proportion of protein models assigned to one of 18,509 OGs for each species. **E** Proportion of OGs specific in each species. **F** Proportion of OGS in orthologous 1–1 relationships and paralogous groupings (1-many, many-1, and many-many) among species compared to *D. v. virgifera*. **G** Number of gene duplication events (dups) at terminal branch points of the species tree. **H** Species tree showing number of gene duplications at internal branch points

Further scaffolding used 431 million 150 bp PE reads from three Dovetail Chicago libraries (table S1), which cumulatively provided a mean physical coverage of ~ 12.11-fold when aligned to the *D. v. virgifera* Dvir_v1.0 assembly (Fig S2a). A HiRise® assembly using these data resulted in a final Dvir_2.0 assembly with 87,712 scaffolds summing to 2.42 Gb (Fig. 1; Table 1). Contiguity increased with a scaffold N50 of 0.489 Mb (Fig S2b) and maximum scaffold length increased to 8.07 Mb; facilitated by reads from library inserts that spanned greater that 300 kb (Fig S2c). Thus, the final Dvir_2.0 assembly is comprised of 585,680 contigs under accession PXJM00000000.2 (PXJM02000001-PXJM02585680) arranged on 87,712 scaffolds (ML014983-ML058324; assembly: GCA_003013835.2; Table 1). Dvir_2.0 retained nearly half a million gaps in 567 Mb of N content. Assessment of representation of arthropoda_odb10 ortholog content in Dvir_2.0 predicted matches to 92.2% (934 of 1013 orthologs). This included 931 (91.9%) complete single copy and 17 (1.7%) complete duplicated. Approximately 5.4% of the lineage orthologs were either fragmented or missing (Table 1; table S4). Compared to other species of Coleoptera from the Family Chrysomelidae having genome assemblies available in NCBI (as of Jan 2022), the number of complete single copy (S) BUSCOs from Dvir_2.0, was similar to the Colorado potato beetle, *Leptinotarsa decemlineata* (Ldec_2.0), and northern tamarisk beetle, *Diorhabda carinulata* (Dcau_1.0). Single copy BUSCOs were greater in Dvir_2.0 than the cowpea weevil, *Callosobruchus maculatus* (Cmac), and ragweed leaf beetle, *Ophraella communa* (Ocom), but the number of fragmented orthologs in Dvir_2.0 were slightly greater compared to all other assemblies (Fig. 2A; table S4).

**Table 1:** Metrics for the *Diabrotica virgifera virgifera* genome assembly, Dvir_v2.0

Metric	Dvir_2.0^1^
Contig assembly	
Total number	586,720
Length (bp)	1,850,691,159
N50 (Mb)	6,227
L50	84,395
N90 (bp)	313,452
L90	1,581
Largest contig (bp)	79,911
Scaffold assembly	
Total number	87,712
Length (bp)	2,418,073,815
GC content (%)	25.2
Gaps	499,008
N bases (within gaps)	567,382,656
N50 (bp)	489,108
L50	1,128
N90 (bp)	37,112
L90	7,527
Largest contig (bp)	8,068,850
BUSCOs (arthropoda_odb10)	
Complete (C)	948 (93.6%)
Complete single copy (S)	931 (91.9%)
Complete duplicated (D)	17 ( 1.7%)
Fragmented (F)	46 ( 4.5%)
Missing (M)	19 ( 1.9%)
Gene Annotations^2^	
RefSeq gene models	25,094
RefSeq mRNA and protein models	28,061
fully-supported^3^	23,405
RefSeq protein-coding gene models	20,592
RefSeq non-coding	4,502
RefSeq pseudogenes	304
Repeat content^4^	53.0%

^1^*Diabrotica virgifera virgifera* WGS Project: PXJM02; GenBank assembly accession: GCA_003013835.2 *(this assembly)*

^2^https://www.ncbi.nlm.nih.gov/genome/annotation_euk/Diabrotica_virgifera_virgifera/100/

^3^> 95% of length with RNA-seq evidence

^4^See RepeatMasker results

### Automated structural gene annotation and orthology predictions

A total of 25,398 RefSeq gene intervals (LOC114324144 to LOC114349541) including 770 tRNAs and 17 rDNA coding regions were predicted in Dvir_2.0 by the NCBI GNOMON pipeline (Table 1; GCF_003013835.1 v100 annotation report). These included 4,502 and 304 non-coding and pseudogenes, respectively. There were 28,061 mRNAs (XM_028127687.1 to XM_028155747.1) putatively transcribed from 20,592 protein coding genes that encoded 28,061 protein models (XP_028127688.1 to XP_028155748.1). Among transcript models, 20,592 (73.4%) were supported by RNA-seq evidence available in NCBI SRA. The largest transcript and gene spanned 60.5 kb and 1.54 Mb, respectively.

A total of 16,458 orthologous groups (OGs) were defined by OrthoFinder among RefSeq models from the fruit fly, *Drosophila melanogaster* (Dmel_6), mosquito, *Anopheles gambiae* (AgamP3), red flour beetle, *Tribolium castaneum* (Tcas5.2), *Anoplophora. glabripennis* (Agla_2.0), *L*. *decemlineata* (Ldec_2.0), and Dvir_2.0 (table S5a). Comparison of OGs among all these species indicated that 6,222 (37.8%) and 8,404 (51.1%) were shared among all six species and all four Coleoptera, respectively (Fig. 2B; table S5b). A species tree based on shared OGs predicted *D. v. virgifera* is most closely related to the three coleopteran species, *L. decemlineata*, *A. glabripennis* and *T. castaneum* (Fig. 2C). Of the 28,061 Dvir_2.0 proteins, 26,456 (94.3%) were assigned to one of 11,221 OGs (2.4 ± 4.4 proteins per OG) (Fig. 2D). Dvir_2.0 protein models contained the greatest proportion of unique species-specific OGs, with exception of the most divergent species, *D. melanogaster* (Fig. 2E). Dvir_2.0 contained the second greatest number of unique OGs (1,230) among the Coleoptera used in analyses (Fig. 2B). The number of one-to-one (1–1) orthologs was greatest between Dvir_2.0 and AgamP3, followed by Dvir_2.0 and Ldec_2.0 (Fig. 2F). The proportions of ortholog classes were similar for Dvir_2.0 compared to Ldec_2.0, Agla_2.0 and Tcas5.2. OrthFinder2 predictions of gene duplication events showed the second greatest number in Dvir_2.0, with the greatest in the most divergent comparator *D. melanogaster* (Fig. 2G), and the greater compared to other Coleoptera (Ldec_2.0, Agla_2.0 and Tcas5.2). The lineage containing all four Coleoptera underwent a total of 1,478 OG duplications from the branch containing *A. gambiae* and *D. melanogaster* (Fig. 2H).

### Manual annotation and gene family curation

**Fig. 3:**
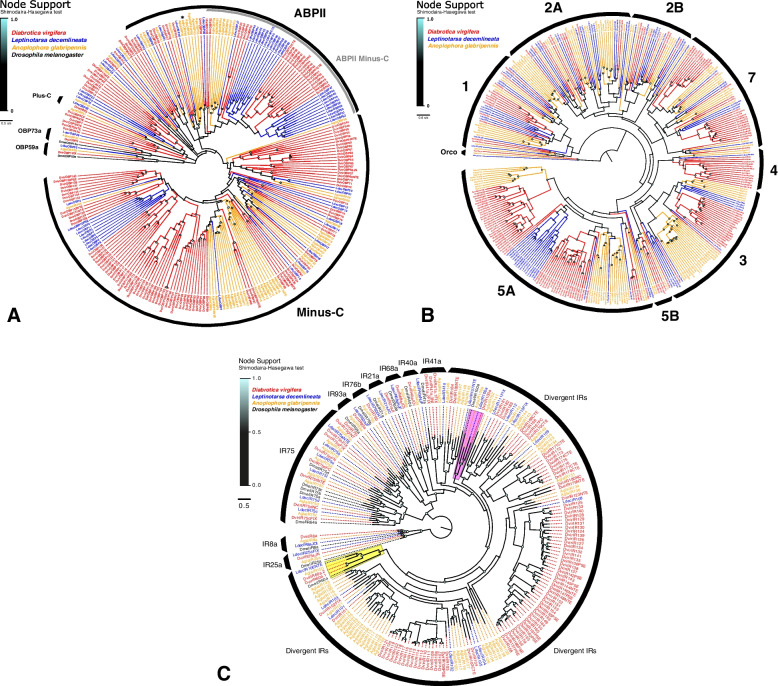
*Diabrotica virgifera virgifera* chemosensory gene family reconstructions shown in phylogenies for **A** odorant binding protein (OBP; unrooted), **B** odorant receptor (OR; rooted by conserved odorant receptor-co-receptor, Orco, lineage), and **C** ionotropic receptor (IR; rooted with the conserved IR8a and IR25a lineages) family members. The clades housing IR60a and IR100a orthologues among the divergent IRs are highlighted in yellow and pink, respectively. Each phylogeny incorporates orthologs from *Diabrotica v. virgifera* (Dvir, red), *Leptinotarsa decemlineata* (Ldec, blue), *Anoplophora glabripennis* (Agla, orange), and *Drosophila melanogaster* (Dmel, black). Bootstrap support at nodes indicated by increasing size and intensity of circles. Black arcs indicate the major recognized subfamilies.

Dvir_2.0 was annotated for three of the largest gene families associated with insect olfactory biology: odorant binding proteins (OBPs), odorant receptors (ORs), and ionotropic receptors (IRs). There were 139 OBP gene models manually annotated from the Dvir_v2.0 genome assembly, along with four pseudogenes (table S6a). The corresponding phylogeny included the five lineages previously documented for beetles [71] (Fig. 3a): each of the three expected orthologs to OBP59a (DvirOBP1), OBP73a (DvirOBP2), and the Plus-C group (DvirOBP3), 50 Classic OBPs (DvirOBP4-53), 31 members of the antennal binding protein II (ABPII) family (DvirOBP23-53), and 90 Minus-C OBPs (DvirOBP54-140) were identified. The Minus-C OBPs included three alternatively spliced genes with two transcript isoforms each (DvirOBP54, 82, and 113), as well as one dimeric gene (DvirOBP107), and one tetramer (DvirOBP128). All three alternatively spliced genes were confirmed via supporting transcriptome data used for the genome annotation (GCF_003013835.1 v100 annotation report). An earlier sequencing effort using ESTs identified 13 of these OBPs, including both isoforms of the alternatively spliced DvirOBP54 [72].

The ORs included the expected single copy odorant receptor co-receptor (Orco) gene, in addition to 151 putatively functional ORs and 10 OR pseudogenes (table S6b). The phylogenetic reconstruction (Fig. 3b) showed a relatively even distribution of ORs across clades that corresponded to all previously described subfamilies [73]. However, *D. v. virgifera* lacked members of Group 6 and included expansions of Groups 4 (DvirOR65-78) and 5A (DvirOR104-161) compared to other chrysomelids (Fig. 3b). Group 2B was resolved as paraphyletic in this phylogeny, lacking one small lineage of ORs that included DvirOR20 (see “2B.iii”) [73].

Overall, only 42 gene models (including Orco) could be extended to their putative full length. An additional 45 genes were nearly complete, missing only 1 or 2 short exons. The remaining models are either missing long or numerous exons, which could be attributed to the fragmented *D*. *v*. *virgifera* genome assembly or large genome size. Manually curated complete OR gene models spanned as much as 100 kb and several incomplete models exceeded 100 kb in length (table S6b). Other models, while apparently full-length, suffered from frequent assembly errors that misplaced or reversed exons within the scaffolds and thus may be chimeric. Putative exons from OR genes which could not be associated with models were noted in 185 additional Dvir_2.0 scaffolds (table S6c).

The IR models included 97 putatively functional genes (75 of these were completed to full-length proteins) and 10 pseudogenes (table S6d). The conserved antennal IRs were difficult to annotate due to the fragmented assembly in combination with many short exons. Among these, exons of 7 genes were interdigitated (in the wrong linear order) on scaffolds and 4 were assembled across multiple scaffolds. Regardless, several IR gene models could be completed by manual annotation based on sequence similarity to conserved orthologs from highly-related species. Specifically, these genes were assigned to the so-called antennal IRs (including DvirIR21a, 25a, 40a, 68a, and several IR41a and IR75 members). Two IR models, DvirIR21aFJ and DvirIR102FIX, were completed manually using raw reads. Twenty additional partial IR genes were composed of single exons assembled on individual scaffolds (table S6d). Genes affected by these assembly issues could not be uploaded as single models in WebApollo, but were instead added as separate parts (indicated within comments). Five individual orphaned exons, likely belonging to the IR75 group as judged from their exon length and sequence homology, were excluded from the final dataset (table S6e)*.* All antennal IRs typical for Coleoptera were identified in *D. v. virgifera*, including genes encoding the co-receptors IR8a and IR25a, with the former being the longest IR gene in the genome spanning > 114 kb of assembled sequence. Phylogenetic relationships among IRs showed one ortholog in *D. v. virgifera* for each of IR76b, IR93a, IR21a, IR40a, and IR68a, but three paralogs of IR41a, and nine genes in the IR75 clade (Fig. 3c).

The repertoire of divergent IRs was particularly expanded in *D. v. virgifera* and included 78 of the putatively functional IRs and 10 pseudogenes. Most of the divergent IRs were encoded by a single exon, although several of these genes were split by one, or in some cases, a few introns. Two lineages of divergent IRs (IR60a and IR100a) are conserved across insect orders [71, 74] and were present in *D. v. virgifera* with one and two members, respectively. Otherwise, only three simple 1:1 ortholog relationships with high support were predicted between *D. v. virgifera* and *L. decemlineata* (Fig. 3c). The remaining divergent IRs have radiated as *D. v. virgifera* species-specific expansions, with one including 43 members (DvirIR124-DvirIR166; Fig. 3c).

A set of 188 unique Dvir_2.0 RefSeq protein models were identified as ABC transporters based on our search criteria (table S7a; mean length of 894.9 ± 415.2 aa; range 199 to 1,640 aa), of which 180 had a putative complete CDS (predicted methionine residue at 1^st^ position and TAA or TAG stop codon). Proteins were encoded by 120 unique genes (1.57 ± 1.34 proteins models per locus; range 1 to 11). BLASTp results showed that 138 Dvir_2.0 RefSeq models matched 64 of the 65 previously identified *D. v. virgifera* ABC transporter protein sequences with *E*-values ≤ 2 × 10^–61^ and identities ≥ 91.1% (2.10 ± 1.64 RefSeq models per protein query; remaining data not shown). Proportional coverage of these queries across Dvir2 RefSeq models ranged from 0.10 to 1.00 (table S7b), and CDS of 12 genes were located on > 1 scaffold and matched intervals of > 1 RefSeq model. This transcript evidence was used to justify joining RefSeq gene and protein models into putative complete CDS (recorded in the Apollo manual annotation tool comments at the i5K Workspace; https://i5k.nal.usda.gov/). Of the remaining 50 putative ABC transporter genes, 31 and 19 were putative full- and partial-length, respectively.

**Fig. 4:**
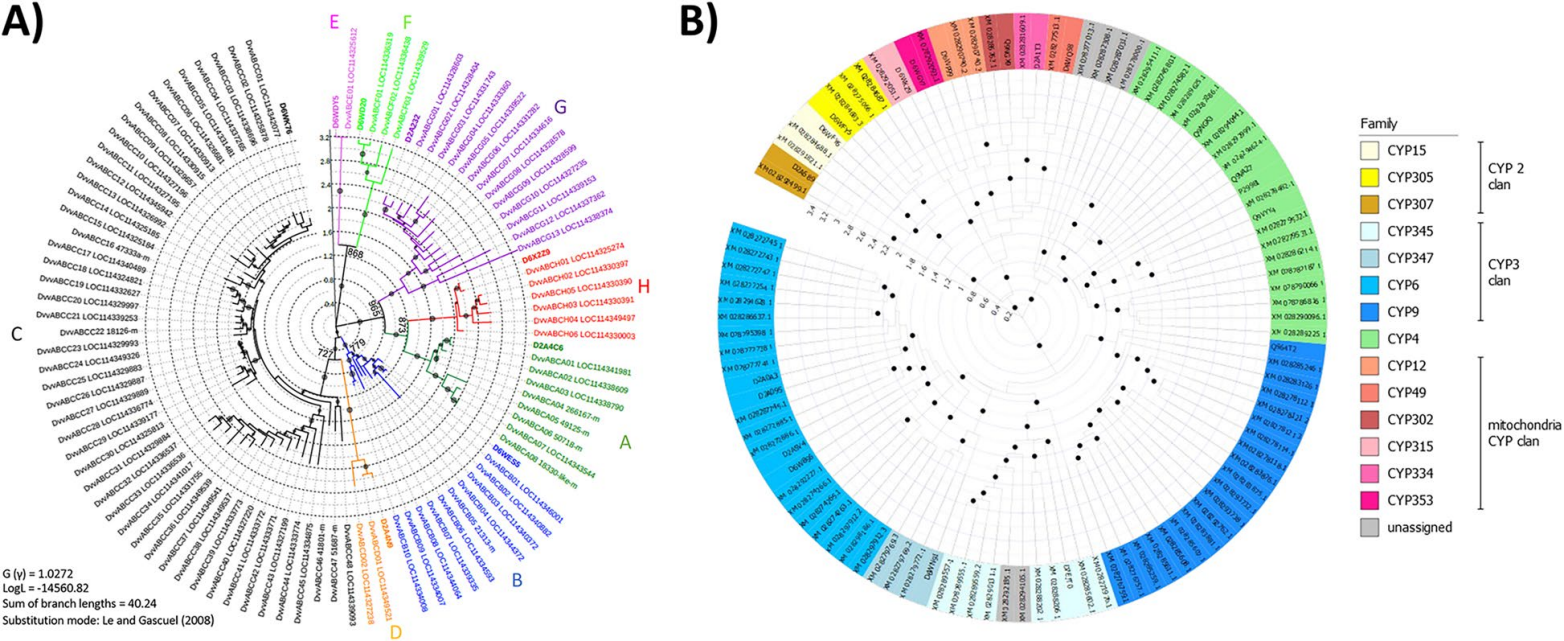
Maximum-likelihood (ML) based phylogenetic reconstructions of *Diabrotica virgifera virgifera* gene families with members putatively involved in *Bacillus thuringiensis* and chemical insecticide resistance. **A** ATP binding cassette (ABC) transporter protein superfamily. Clades corresponding to subfamilies A to H are indicated with respective colors as defined by included *Tribolium castaneum* orthologs (bold). **B** Cytochrome P450 (CYP) gene families: Color coded members are indicated as defined by *T. castaneum* orthologs (bold). Node support of ≥ 500 from 1,000 bootstrap iterations are indicated by dot size on a 10-point scale and shown numerically for nodes separating subfamilies. Internal scale indicates number of amino acid changes per site from among retained positions within corresponding amino acid alignments

Ninety-three *D v. virgifera* ABC transporters were assigned to one of 8 subfamilies based on phylogenetic predictions from a multiple protein sequence alignment of the complete nucleotide binding domain 1 (NBD1) (table S7c). The BIC score was maximized at 18,151.2 for the LG model of protein sequence evolution and applied along with an empirically derived gamma parameter (G) of 1.0272. The resulting ML tree topology consisted of 8 clades, each corresponding to subfamilies A-H as defined by *T. castaneum* orthologs (Fig. 4a). Bootstrap node support from ≥ 727 of 1,000 random trees was obtained for nodes separating subfamilies, but node support within subfamily failed to surpass 500 in many instances. The number of *D. v. virgifera* ABC transporters per subfamily ranged from 1 for subfamily E to 48 within subfamily C.

One hundred and eighty-one *Diabrotica* RefSeq models were identified as cytochrome P450 (CYP) monooxygenases. Of these, 85 were determined to be full-length and subsequently assigned to 30 different clusters with ≥ 40% identity (table S8a). Three RefSeq models XM_028278121.1, XM_028297912.1, and XM_028279769.1 were determined to be concatenated protein coding sequences and subdivided into two component CDS. Queries of the UniProt KB database with a representative member of each cluster resulted in assignments to 14 CYP families, along with 6 unassigned due to “hits” below cutoff thresholds (table S8b). Based on these results, a representative sequence was assigned to 24 of 30 clusters (80.0%), with 17 from *T. castaneum*. This cluster assignment led to placement of most of the 85 P450s into families CYP4 (20%; *n* = 17), CYP6 (22%; *n* = 19), and CYP9 (22%; *n* = 19), with the rest assigned to 1 of 11 other families (*n* = 24) or remained unassigned (*n* = 6). The subsequent ML-based phylogenetic reconstruction using an alignment of 85 full-length CYP sequences along with representative sequences from other insects defined membership of *D. v. virgifera* orthologs to 14 CYP families (Fig. 4b). Specifically, all 85 full-length Dvir_2.0 CYP members were putatively placed within 14 families, with the exception of two sequences that were not assigned but fell within the clade containing CYP 354 family members.

### Differential gene expression in response to larval host plant feeding

**Fig. 5:**
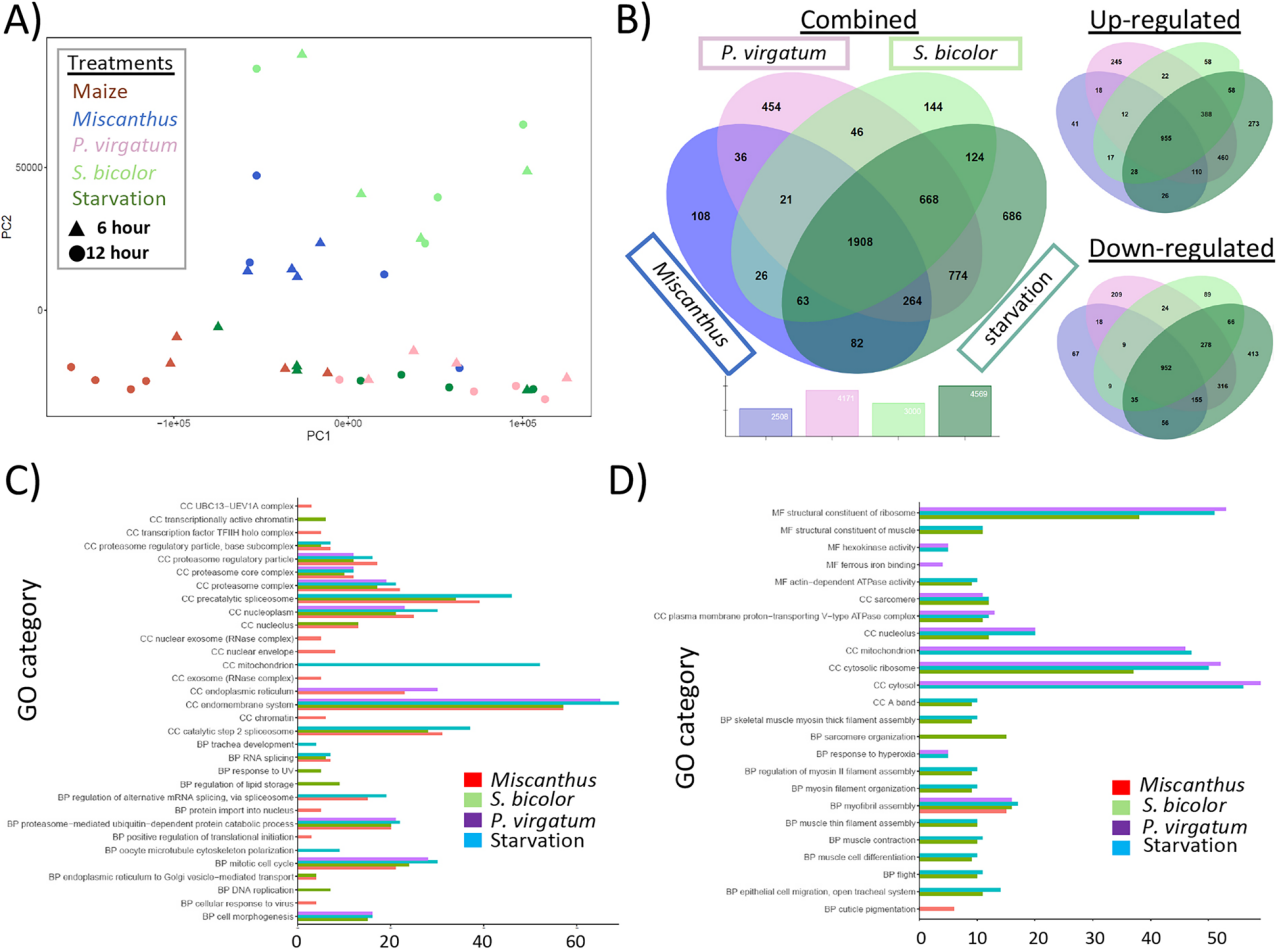
Differential expression of transcripts between *Diabrotica virgifera virgifera* larvae feeding on different host plants. **A** Principal component analyzes (PCA) of transcript expression levels between *D. v. virgifera* larvae feeding on different host plant at 6- and 12-h time points (*n* = 40 treatments). **B** Differentially-expressed transcripts shared among larvae feeding on maize roots compared to exposures on alternate host (*Miscanthus*), marginal host (*Panicum virgatum*), poor host (*Sorghum bicolor*) and starvation treatments. **C** Gene ontology (GO) terms significantly enriched among transcripts up-regulated in *D. v. virgifera* feeding on maize roots compared to other host plant exposures and a starvation treatment. **D** Significantly enriched GO terms among transcripts down-regulated in *D. v. virgifera* larvae on maize compared to other treatments

A total of 524.4 million Illumina reads were generated in RNA sequencing (RNA-seq) data, with mean number of reads among four replicates for each 6-h maize, switchgrass, *Miscanthus*, *Sorghum bicolor* and starvation treatment ranging from 10.7 ± 1.4 to 13.6.9 ± 0.4 million (table S9). The number of reads among the corresponding 12-h treatments ranged from 13.0 ± 0.6 to 14.0 ± 2.0 million. Analyses of read counts within pseudo alignments to Dvir_2.0 RefSeq transcript models predicted 30 to 891 differentially-expressed genes (DEGs) between exposure times within maize, *Miscanthus*, *P. virgatum*, *S. bicolor*, and starvation treatments (Table 2a; along diagonal). These comparisons also showed 93 to 3,858 significant DEGs between treatments across all exposure times, where maize 12-h compared to *Miscanthus*, *P. virgatum*, *S. bicolor*, and starvation at 6-h showed greater numbers of DEGs. Specifically, within the 6-h treatment times, *Miscanthus* showed 1089 DEGs compared to maize which was lower than in the comparisons involving *S. bicolor* (*n* = 1103), *P. virgatum* (*n* = 2511) and starvation (*n* = 2945). The number of DEG for maize compared to *Miscanthus* fed larvae at 12-h (*n* = 3077) were ≥ 17.2% lower than the numbers estimated in exposures to maize compared to *P. virgatum* (*n* = 3858) or S. *bicolor* (*n* = 3715). Between host plant treatments within 6- and 12-h exposure, there were 0 to 1,691 and 16 to 4,569 predicted DEGs, respectively (Table 2b), where the lowest number of DEGs were predicted in maize compared to *Miscanthus* across treatment times than for comparisons with other host plants. The number of DEGs was lowest between *S. bicolor* and *Miscanthus* at both exposure times. The greatest number of DEGs in both 6- and 12-h exposures were between maize and starvation treatments, but larvae on maize compared to *P. virgatum* had the greatest number of differences in comparisons that involved plant exposure. The greatest overall number of DEGs were predicted between maize and other treatments at 12-h: starvation (*n* = 4,569; 2,298 up-regulated/2,271 down-regulated); *P. virgatum* (*n* = 4,171; 2,210/1,961); *S. bicolor* (*n* = 3,000; 1,538/1,462); and *Miscanthus* (*n* = 2,508;1,207/1,301; table S10). Principal coordinate 1 (PC1) and PC2 accounted for most of the total variation read counts among replicates within and between treatments, respectively (Fig. 5a; distinct clusters were observed for larvae fed maize, *Miscanthus*, and *S. bicolor*). There were 1,908 DEGs shared across all treatments (955 and 953 up- and down-regulated, respectively), and starvation and *S. bicolor* treatments shared the greatest number of DEGs (Fig. 5b).

**Table 2:**
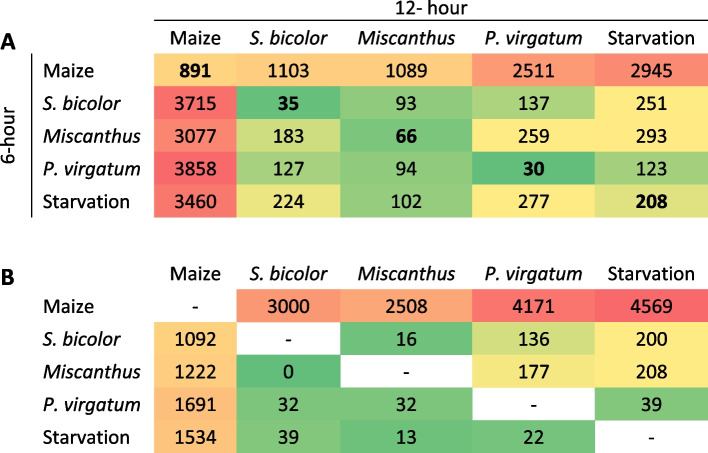
Predicted number of significant differentially expressed transcripts between *Diabrotica virgifera virgifera* larvae exposed to roots of maize (primary host), *Miscanthus* (*Miscanthus* x *giganteus*; alternate host), and *Panicum virgatum*; marginal host), and *Sorghum bicolor* (poor host) or starvation conditions. Comparisons among treatments **A** between exposure times among plant treatments (Comparisons within plant treatment between time points are in bold), and **B** across plant treatments within exposure time (6- and 12-h exposures below and above diagonal, respectively)

Initial BLASTp searches of the GOanna invertebrate portion of the UniProt_KD database yielded 8,316 “hits” to 6,820 Dvir_2.0 protein models (*E*-values ≤ 10^–40^; % identities ≥ 40.02). Parsing the 50,416 associated GOs identified those with putative function in biological process (BP; *n* = 33,397), molecular function (MF; *n* = 9,190) and cellular component categories (CC; *n* = 11,027) (GOanna and GOslim data available Ag Data Commons) [75]. Enrichment analysis of GO terms assigned to proteins encoded by transcripts significantly up-regulated in the maize treatment at 12-h compared to other plants after 6-h were mostly associated with transcription, protein breakdown, and development (data not shown). The DEGs predicted among neonates on maize compared to other plant treatments at 12-h was further analyzed to identify host-specific responses after more prolonged exposure. These comparisons within exposure time were made to avoid confounding factors influencing expression based on duration of feeding. GO terms assigned to transcripts significantly up-regulated in *D. v. virgifera* neonates after 12-h exposure to maize were significantly enriched in 18 CC categories (Fig. 5c). Among these, the greatest number were associated with the “endomembrane system” for comparisons to *Miscanthus*, *S. bicolor*, *P. virgatum*, and starvation treatments. “Endoplasmic reticulum (ER)” had the greatest number of CC terms for differences not involving starvation. Multiple categories associated with “proteosome” were over-represented among transcripts up-regulated in maize compared to the four other treatments. Enrichment of “mitochondrion-localized products” were predicted only for maize compared to the starvation treatment, and two spliceosome-associated categories were enriched in all comparisons except *P. virgatum*. GO terms for BP “mitotic cell cycle” and “proteosome-mediated ubiquitin-dependent protein catalysis” were most prevalent between maize and *Miscanthus*, *S. bicolor*, *P. virgatum*, and starvation treatments (Fig. 5c).

Few GO terms were significantly over-represented among transcripts down-regulated in maize compared to *Miscanthus* (Fig. 5d). The MF and CC terms “structural constituent of ribosome” and “cytosolic ribosome”, respectively, were identified among transcripts down-regulated in maize compared to *P. virgatum*, *S. bicolor*, and starvation. The CC category GO terms “mitochondrion” and “cytosol” were significantly enriched for maize comparisons to *P. virgatum* and starvation treated larvae. “Myofibril assembly” was the only BP term associated with transcripts down-regulated in maize compared to all plant and starvation treatments. Several BP categories associated with muscle assembly, differentiation and action were enriched among transcripts down-regulated in larvae on maize compared to those on *P. virgatum* or starved. Although GO categories associated with cytochrome P450s were not significantly enriched, 50 RefSeq gene models annotated as P450s were among the DEGs (table S10; 39 and 11 up- and down-regulated, respectively), with 94% belonging to clans 4 (*n* = 13), 6 (*n* = 21) and 9 (*n* = 13). Among these 22 were shared among all comparisons after 12-h exposures, with cytochrome P450 9e2-like (LOC114344243) mRNA being the most highly up-regulated in all comparisons [Log2(fold-change) 2.158 to 2.819]. Starvation and *P. virgatum* exposed larvae shared the greatest number of differentially-expressed P450s (*n* = 34), and 2 to 6 were unique to comparisons.

## Discussion

Annual costs associated with *D. v. virgifera* control and reduced crop yields in the United States are estimated at over $2 billion [76]. Our draft genome assembly is the first published for *D. v. virgifera* and serves as a resource for investigating factors influencing the adaptability of this species to changes in the agroecosystem. The 2.56 Gb flow cytometry-based *D. v. virgifera* genome size [69] is among the largest among beetles, compared to 66 from coleopteran species that range from 0.154 to 2.578 Gb [77], and it is the second largest yet described among species in the Chrysomelidae [78]. Genome assemblies previously reported for six chrysomelids are all smaller than Dvir_v2.0 (0.654 to 1.731 Gb; table S2). Despite the large *D. v. virgifera* genome size and use of short read data for assembly, Dvir_2.0 shows the third highest scaffold N50 (489 kb; Fig. 1). Additionally, Dvir_v2.0 has a high representation of complete BUSCOs in arthropoda_odb10 (93.6%; 804 single copy and 198 duplicated), but also the greatest proportion of fragmented BUSCOs (4.6%) compared to other coleopteran assemblies. The latter may be a consequence of the fragmented Dvir_2.0 assembly, likely resulting from a combination of using short reads for assembly [79] and the highly repetitive nature of the *D. v. virgifera* genome [69, 80, 81] (Fig S1). Thus, the length and low sequence divergence among repeat classes may have posed difficulties in assigning unique placement of reads into assembled contigs. Attaining highly contiguous assemblies remains a challenge for many species with large or complex genomes due to abundandace of recently expanded repeats and transposable elements [82, 83]. Such fragmented assemblies can occur even when using long single molecule reads [84]. Ironically, the high number of repeats in the *D. v. virgifera* genome likely contributed to Dvir_2.0 being a poor resource for estimating their abundance, due to contribution to the nearly 0.5 Gb of gaps (N bases). Therefore, any analyses of repeat content were not undertaken for Dvir_2.0 in lieu of future and potentially more complete assemblies. Nevertheless, Dvir_2.0 provides a resource for the unbiased system-wide identification of genes in pathways of biological or physiological importance, including those putatively involved in host plant attraction and adaptation. Understanding the mechanisms underlying these aspects is crucial to opening gateways for developing novel pest insect control strategies [85–87].

Chemosensory pathways in *D. v. virgifera* are involved in perception of plant volatiles attracting larvae to roots [88, 89], adults to oviposition [90] and feeding sites [91], and sexual communication [92, 93]. OBPs are soluble proteins present in the sensillar lymph that were initially believed to transport odorants to the chemoreceptors, but now are recognized as playing diverse olfactory and non-olfactory roles [94–99]. ORs of beetles comprise exceptionally large radiations of chemoreceptors and are the primary means by which volatile compounds are recognized by the olfactory system [73]. IRs are presumed to be the ancestral mode of olfaction in insects, and they are categorized into two main groups: “antennal IRs” that are broadly conserved across the Insecta and contribute to olfaction (acids, nitrogen-containing compounds, and aromatics [99–101] and “divergent” IRs that are highly variable across species and presumed to function in taste [102–104].

The 139, 151, and 75 putatively functional OBPs, ORs, and IRs, respectively, identified in Dvir_2.0 suggest varying degrees of copy number expansion (Fig. 3), where *D. v. virgifera* OBP and IR families show the greatest proportional increase compared to other chrysomelids [71, 104]. In fact, the genome includes considerably more OBPs than any other insect species annotated to date [105–109], eclipsing even the 109 OBP genes of the German cockroach, *Blattella germanica* [110]. OBPs carry out a diversity of functions including chemoreception [108], and thus these changes could support prior evidence that OBP repertoires change more rapidly in specialist species [111]. However, the primary radiation of OBPs was in the family of Minus-C family, which may be non-olfactory in beetles [112]. Thus, this expansion may ultimately be unrelated to the chemical ecology of *D*. *v*. *virgifera*.

Our data also confirm that a second radiation of Minus-C proteins independently evolved in chrysomelids, as previously suggested based on data from *L*. *decemlineata* [113]. This novel radiation of Minus-C genes emerged within the ABPII family, and it is present in both the Cerambycidae and Chrysomelidae (Fig. 3a; “ABPII Minus-C”). No ABPII Minus-C proteins are present in the related phytophagan *Dendroctonus ponderosae* nor the more distant cucujiform *T*. *castaneum* [71], further establishing this as an independent event in the superfamily Chrysomeloidea. This emphasizes the fluidity with which insect OBPs switch between a four and six cysteine state, which has also occurred separately in Diptera and Hymenoptera [105]. However, the underlying drivers and functional consequences of such a switch remain unknown, and should be investigated in future studies.

Among the chemoreceptors, the largest proportional expansions were among the ORs within Group 5A (58 genes) and Group 4 (14 genes) compared to the chrysomelid *L. decemlineata* and the cerambycid *A. glabripennis*. Members of either OR subfamily are yet to be functionally characterized, although 5A is typically expanded in cucujiform beetles, and expression analyses in other species suggest an association with diet and mouthparts [108]. In any case, due to the systemic assembly errors that affected annotation of this gene family, we strongly caution that our OR models be taken as a preliminary effort, and resequencing will be necessary to fully resolve the gene family. The 107 annotated IRs in *D. v. virgifera* exceeded the range of 27 to 81 genes in other coleopterans [71, 113], which is driven by the particularly large expansion of the species-specific divergent IRs (88 genes). This again suggests a diversification of gustatory rather than olfactory function [100]. Our annotation focused exclusively on olfactory proteins and therefore did not include the insect gustatory receptor family (GRs), but we expect that any future annotation will reveal correspondingly large expansions.

ATP-binding cassette transporters comprise one of the largest gene families wherein subfamilies A-H in arthropods function in a variety of transmembrane transport activities [114], including involvement in development [115] and detoxification [116], and potentially in Bt resistance mechanisms. There are 188 complete and partial RefSeq annotated ABC transporters in Dvir_2.0, which is greater than a transcriptome-based estimate of 65 from a prior *D. v. virgifera* transcriptome-based estimate [117]. Phylogenetic reconstruction using 93 Dvir_2.0 models containing a full-length NDB placed 10 members into an ABCB clade, suggesting the number of orthologs are nearly double that predicted in *T. castaneum* and equal to that in *A*. *viridicyanea*. This subfamily includes an ABCB member near a locus controlling *D. v. virgifera* Bt Cry3Bb1 resistance [118]. This subfamily may be important in that heterologous expression of *D. v. virgifera* ABCB1 mediates Bt Cry3Bb1 toxicity [119]. Our phylogenetic reconstruction also suggests expansion of the 49 member *D. v. virgifera* ABCC subfamily (Fig. 4a) compared to 35 in *T. castaneum* [115] and 37 in *A*. *viridicyanea* [104]. ABCC subfamily members are involved in a variety of transport and receptor functions [114], and in phase II detoxification of xenobiotics via efflux of insecticides or their metabolic products [120]. Regardless, differential regulation of ABC transporters is not associated with *D. v. virgifera* resistance to organophosphate [65] or pyrethroid insecticides [121]. Interestingly, although only 17 ABC transporters were among the DEGs across all treatment comparisons to maize at 12-h exposure, 15 (88.2%) were ABCC members. Functional characterization of these genes will be required to determine any importance in response to consumption of different plant materials, or general stress responses of *D. v. virgifera* as hypothesized earlier [64].

Our corresponding reconstruction of the *D. v. virgifera* cytochrome P450 gene family was particularly problematic due to a high proportion of RefSeq models having partial coding sequences. Nevertheless, we show that CYP4, CYP6, and CYP19 clades have the greatest number of orthologs among full-length *D. v. virgifera* models (Fig. 4b). Members of these clades are associated with insecticide detoxification in *Diabrotica* [65, 121] and other arthropods [122]. Many of these clades also show functional overlap with potential involvement in metabolic adaptation to host plant defenses [123]. Incorporation of Dvir_2.0 gene models from among the 95 partial ABC transporters and other cytochrome P450s not included in our phylogenetic classifications will undoubtedly lead to prediction of even more expansive repertoires and allow future elucidation of function. Interestingly, detoxification was not among the over-represented GO terms associated with transcripts up-regulated following 12-h exposures to maize compared to other plant exposures, in contrast to predictions in other insect species [15, 124–128]. Regardless, 50 P450s were predicted with a majority being up-regulated (78%) or putatively belonging to clan CYP6. Interestingly, 22 were shared in comparisons of maize with other plant exposure and starvation treatments, where such shared expression was previously attributed to general *D. v. virgifera* stress responses [64]. The arthropod cytochrome P450 family is comprised of 6 clans involved in oxidation of a variety of endogenous and exogenous substrates [128, 129]. Differentially-expressed P450s in the CYP6 clan were generally associated with insecticide resistance compared to the CYP3 clan where members are responsive to phytochemicals [129]. As with most insects, functional annotation data remain sparse for *D. v. virgifera*, and assignment of specific functions to host plant or starvation responses remain speculative.

The most prevalent GO CC terms among up-regulated transcripts across all comparisons of larvae fed maize to those on alternate hosts or starved (Fig. 5c), endomembrane transport and nucleoplasm, implicate structures coordinating intracellular transport, and/or transcriptional regulation, [130, 131]. Specifically, the endomembrane system includes the interconnected endoplasmic reticulum, Golgi, nuclear envelope, lysosomes, and various transport vesicles, many of which are involved in conserved eukaryotic stress responses [132]. Our predicted increase of proteosome and lipid catalysis activities in maize-exposed *D. v. virgifera* larvae represent other highly conserved functions associated with modulation of cellular stress response across eukaryotes [133–135]. These functional categories were also up-regulated in the maize adapted European corn borer, *Ostrinia nubilalis*, compared to the mugwort specialist *Ostrinia scapulalis* when feeding on maize [124]. This evidence could suggest the importance of maintaining homeostasis or remediating cell stress, as was concluded for a study comparing *Drosophila* feeding on preferred and non-preferred hosts [125]. This up-regulation of proteosome and lipid catalysis activities may also be connected with the elevated activity of stress response pathways, initiating protein degradation across eukaryotes [135]. Alternatively, some pathways may be indicative of growth, development and related cellular processes. Given evidence of greater survival rates of *D. v. virgifera* larvae on maize roots compared to less preferred hosts [22–24, 27], an equilibrium between pathways involved in growth and stress responses may represent adaptations “optimized” for feeding on maize. Regardless, there is an overall dearth of knowledge regarding transcriptional changes in response to host plants or specific defensive compounds, and the observations made here require addition investigations.

Compared to larvae feeding on maize, those fed *Miscanthus* showed fewest DEGs compared to those on other host plants after 12-h (Table 2a) and had only two significantly enriched GO terms among transcripts that were down-regulated. Analogous results of lower numbers of DEG among larvae on *Miscanthus* compared to maize than all other host plant exposures were found among the 6-h treatment time (Table 2a) as well as comparisons across treatment times (Table 2b). Furthermore, PCA demonstrated that larval transcript abundances within maize and *Miscanthus* treatments roughly clustered in the lower left quadrant of PC1 and PC2. Given *Miscanthus* shares a more recent common ancestry with maize than *S. bicolor* [136] and our results showing the fewest observed shifts in gene regulation compared to the preferred maize host, might support the premise that insect herbivory on closely-related host plants may require accumulation of a few physiological adaptations [137]. Although the number of distinct GO terms were greatest for feeding on maize compared to *S. bicolor* and starvation, the terms with the most down-regulated transcripts were present in maize comparisons to *P. virgatum*, *S. bicolor*, and starvation. These patterns could indicate differences in levels of feeding. Specifically, *S. bicolor* and *P. virgatum* do not support larval *D. v. virgifera* growth and development or adult emergence [22–24], so overlap of putative functional categories with starvation may reflect lack of feeding. Acute starvation enacts a diverse set of highly conserved cell responses that engage internal energy reserves, cannibalize cell constituents, and suppress energy-dependent processes such as mitochondrial ATP production and translation [138]. These processes may be reflected in the significantly enriched mitochondrial and ribosomal components and functions among predicted significantly down-regulated transcripts in larvae exposed to *P. virgatum*, *S. bicolor*, and starvation treatments compared to maize. Additionally, changes in myosin interactions with the actin cytoskeleton [139] and reduction of intracellular vesicle transport are associated with starvation [140, 141]. This offers an enticing explanation for the array of down-regulated muscle-related processes (myosin, myofibril, and sarcomere) enriched in *S. bicolor* and starvation treatments compared to maize. By comparison, similar differences were not predicted between maize and *Miscanthus*, suggesting co-adaptation to both these hosts and supported by observations that *Miscanthus* also sustains *D. v. virgifera* development [46].

Polyphagous insects are expected to evolve a larger repertoire of digestive and defensive responses than specialists which will comparatively evolve narrower and more optimized responses [141, 142]. The former tends to utilize transcriptional plasticity in response to dietary changes [143], suggesting the rather limited variation in putative functions carried out among up-regulated *D. v. virgifera* genes in response to different hosts may reflect a correspondingly restricted transcriptional plasticity. The apparent lack of significant changes or enrichment of detoxification pathway components in *D. v. virgifera* larvae is in stark contrast to analogous prior experiments in other insects [15, 124, 125, 127], including the maize preferring lepidopteran insect, *O. nubilalis* [124]. Differences with prior studies could be a consequence of exposure times, type and level of maize defenses in vegetative (leaf) compared to more vascular tissues (roots), or factors affecting rates of transcriptional response. Alternatively, lack of host suitability and potential for impacts of reduced larval feeding in *S. bicolor* and *P. virgatum* treatments may have caused starvation as opposed to defense responses in *D. v. virgifera* larvae. Over interpretation of changes in GO categories and putative gene functions among DEGs should be cautioned due to lack of function validation, but overall trends in these changes may support the broad conclusions presented in this study.

## Conclusions

The current study demonstrates the species-specific expansion in the number of chemosensory and ATP transporter family members, and dynamics of differential gene regulation in response to host plant exposures in *D. v. virgifera*. The latter arguably might be a consequence of a long-standing co-evolutionary relationship. Maize domestication began ~ 9000 years ago in southern Mexico [144], which altered the maize progenitor, teosinte, *Zea diploperennis*, to create plants suited for grain production [145]. The reduction in genetic diversity during domestication resulted in decreased native resistance to *D. v. virgifera* root feeding [146], or conversely coincided with the adaptation of *D. v. virgifera* to maize root defenses. Adult *Diabrotica* are found in fields of *Z. diploperennis* and feed on its roots [147]. This suggests that in addition to the premise that *D. v. virgifera* adapted to maize during crop domestication [18], maize plant defenses and insect countermeasures may have evolved from more ancestral relationships. Interestingly, this relationship resulted in the ability of *D. v. virgifera* larvae to sequester maize benzoxalzinoid defensive compounds and repurpose them for their own nematode and bacterial pathogen defenses [148]. This high degree of host specialization and multiple adaptations to control practices by *D. v. virgifera* apparently contradicts the pre-adaptation hypothesis [149]. The “pre-adaptation” of an insect species is purported to result from a series of adaptations to a broad range of host plant defenses that also leads to increased survival when exposed to insecticidal agents, wherein adaptations may include the capacity to up-regulate detoxification and metabolic functions [150–152]. Thus, ancestral *D. v. virgifera* adaptations to host plants might have evolved to a diversity of teosintes as well as genetically diverse maize land races, each with various host plant resistance traits [153]. Population, ecological or genomic scale and other biotic or abiotic factors may lead to the reoccurring and relatively rapid adaptations to control measures by this species. Whether these adaptations by *D. v. virgifera* result from selection on extant or de novo mutations are unknown. Further population and functional genomic studies using our genome assembly and other developing resources will undoubtedly shed light on the ability of *D. v. virgifera* to continually adapt to a changing agroecosystem.

## Materials and Methods

### Genome sequencing and scaffold assembly

Individuals from a Bt-susceptible non-diapausing strain of *D. v. virgifera* [154], maintained at the United States Department of Agriculture, Agricultural Research Service (USDA-ARS), North Central Agricultural Research Laboratory (NCARL), were previously used to estimate a genome size of 2.58 Gb (2.80 pg) [69]. An inbred line, Ped12, was generated from this non-diapausing strain by an initial mate pair from which inbreeding was continued by a single pair full-sib mating in generations 1 (G_1_) through G_5_, full-sib mating *en masse* in G_6_ and G_7_, and then single pair full-sib mating in G_7_ to G_9_. Ped12 was then maintained *en masse* for six additional generations post inbreeding (Ped12-6) with a constant size of ~ 1,000 individuals prior to use in this study.

Although no *D. v. virgifera* karyotypes are available, females in other *Diabrotica* are homogametic (XX) and males hemigametic (XO) [155, 156]. To maintain equal depth of coverage across the X chromosome, sequencing libraries were prepared from adult females. DNA was extracted from three adult Ped12-6 females (BioSample SAMN08631342; sample Ped12-6-A-3) using a modified sodium dodecyl sulfate (SDS) method. For this, single or pooled adult females were ground in liquid nitrogen, incubated overnight at 50 °C in extraction buffer (0.2% SDS, 150 mM NaCl, 100 mM Tris/HCl, 25 mM EDTA, pH 8.0) with 1.0 mg ml^−1^ Proteinase K, and then treated with RNaseA at 37 °C for 2-h. Proteins and debris were removed by high-salt precipitation overnight and centrifugation at 4 °C. DNA was then ethanol precipitated, air dried, resuspended in 10.0 mM Tris, pH. 8.0, and quality checked by 0.9% agarose gel electrophoresis and quantified using Qubit dsDNA BR Assay Kit (Invitrogen, Waltham, MA, USA). Approximately 2.0 µg from each extract was submitted to the DNA Services Lab at the Roy J. Carver Biotechnology Center, University of Illinois, Urbana, IL. Genomic DNA from a single female (#1; table S1) was used to construct two PE libraries with ~ 0.5 kb inserts using TruSeq DNA Library Prep Kits v1 (Illumina, San Diego, CA, USA). A separate female (#2) was used to construct an ~ 1.5 kb PE library. MP libraries were constructed using the Illumina Mate Pair Library v1 Sample Prep Kits v1 (Illumina) from an equimolar pool of DNA from two different adult females (#3 and #4) and size selected for ~ 5.0 kb and 10 kb insert MP libraries (table S1). An additional 15.0 kb MP library was prepared from a pool of four females using the Nextera Mate-Pairs Sample Prep Kit (Illumina). Paired-end 100-bp sequence reads were generated for all PE libraries in a total of 10 lanes of a HiSeq2000 (Illumina). Each MP library was similarly sequenced on one or two lanes with 50- or 100-bp read lengths.

FASTQ reads were trimmed to remove Illumina adapters and sequence with a Phred quality score (*q*) < 20 using Trimmomatic [157] or the FASTX Toolkit (http://hannonlab.cshl.edu/fastx_tookit/). A sum of canonical k-mer counts at *k* = 31 was made from 0.5 kb insert PE reads using Meryl [158] and the k-mer histogram used to estimate genome size, heterozygosity, duplication level and other parameters with the R script, GenomeScope 2.0 [159]. MP libraries were filtered to remove redundancy and retain mates of appropriate distance and orientation using custom scripts. Trimmed reads were then error-corrected by library with Quake [160] by counting 19-mers. Surviving 0.5 kb PE reads were used as input for the SOAPdenovo assembler v 2.04 [161] with a k-mer parameter set to 49 (K = 49) for contig assembly. The 1.5-bp insert PE and MP libraries were progressively ranked and used by SOAPdenovo for iterative scaffolding. Gaps were closed with the 0.5 kb PE library read data using GapCloser v1.10 [162].

The unordered genomic contigs generated by SOAPdenovo were joined based on sequential gapped alignments with two transcript assemblies. The first round of transcript-guided scaffolding used 87,996 transcripts assembled across different growth stages (NCBI TSA accession GHNJ00000000.1 [117] to query against the *D. v. virgifera* SOAPdenovo GapCloser assembly with the BLAST-like alignment tool (BLAT) [163]. Searches used default parameters, except that the resulting.psl file header was suppressed (-noHead) and minimum sequence identity (-minIdentity) was set at 0.95. Output was filtered for single best hit score (–best) spanning a given genome interval with a minimum identity (–minId) ≥ 95 and minimum coverage (–minCover) ≥ 70 with the script filterSPL.pl (http://augustus.gobics.de/binaries/scripts/filterPSL.pl). The filtered.psl file and the *D. v. virgifera* SOAPdenovo GapCloser genome assembly were used as input for an initial run of L-RNA-Scaffolder, an application that determines order and orientation of fragmented contigs encoding partial gene CDS based on long transcript sequence assembly data [70], using default parameters. The resulting scaffolded and non-scaffolded fasta sequences were joined using fastaConcat.pl (http://raven.iab.alaska.edu/~ntakebay/teaching/programming/perl-scripts/perl-scripts.html).

In a second round, the BLAT query and L-RNA-Scaffolder steps were performed as described above, except queries were made with 116,070 transcripts from a *D. v. virgifera* reference transcriptome (TSA accession ERZ1775117.1) [64], and the merged set of FASTA data from round 1 were used as the BLAT database. The resulting round 2 scaffold and non-scaffolded FASTA output were again joined.

Redundant sequences (independently assembled haplotypes putatively derived from the same genomic location) were identified and removed from the transcript-guided assembly using Redundans [164]. Redundant sequences were defined as two or more sharing ≥ 95% similarity over ≥ 90% of a given length. The algorithm randomly assigns one haplotype to represent the genome region within the final assembly. Sequence contaminants were identified with BlobTools [165] and custom vector screening scripts, and then filtered from the assembly. The resulting set of contigs were submitted to National Center for Biotechnology Information (NCBI) as a whole genome shotgun (WGS) under accession PXJM00000000.1 (sequence range PXJM01000001-PXJM01579519; *n* = 579,519) along with scaffolds (KZ688668-KZ772672) to constitute assembly Dvir_v1.0 (assembly accession GCA_003013835.1, depreciated; Fig. 1).

### Scaffolding

Adult Ped12-6 females were provided to Dovetail Genomics (Scotts Valley, CA, USA), where three custom “Chicago” libraries were constructed as described previously [166]. In brief, chromatin was reconstituted in vitro from ~ 500 ng of high molecular weight DNA with mean length of 100 kb and then formaldehyde fixed (crosslinked). Following digestion with *Dpn*II, 5’ overhangs were blunted by filling in with biotinylated nucleotides and ligated. Chromatin crosslinks were then abrogated, followed by DNA purification, and final shearing to ~ 350 bp mean fragment sizes. Libraries were constructed using the NEBNext Ultra Library Prep kit (New England BioLabs, Ipswich, MA, USA) with Illumina compatible adapters, and each library sequenced for 150 cycles on an Illumina HiSeq X (Illumina, San Diego, CA, USA). Subsequent scaffolding used the “HiRise” software pipeline as described earlier [166], wherein PE “Chicago” library reads were first aligned to the Dvir_v1.0 assembly (Fig. 1) using a modified version of the Scalable Nucleotide Alignment Program (SNAP; https://www.microsoft.com/en-us/research/project/snap/). Likelihood models were used by HiRise to estimate distances separating PE Chicago library reads mapped to Dvir_v1.0, and then formed prospective joins and corrected putative misjoins. Completeness of the scaffolded assembly was assessed using the Benchmarking Universal Single-Copy Orthologs (BUSCO) tool v.5.2.2 with 1,013 orthologs in the Arthropoda_odb10 gene set [167–169]. This assembly, Dvir_2.0, was submitted to NCBI (assembly GCA_003013835.2; Table 1), which constituted 585,680 contig sequences in PXJM00000000.2 (sequence range contigs: PXJM02000001-PXJM02585680 PXJM01000001-PXJM01579519) and 87,712 scaffolds (ML014983-ML058324).

### Automated structural gene annotation and orthology predictions

*Ab initio* and evidence-based gene predictions for Dvir_2.0 were made using the Gnomon eukaryotic genome annotation tool [170] as performed by NCBI automated eukaryotic genome annotation pipeline v 8.1 [171] https://www.ncbi.nlm.nih.gov/genome/annotation_euk/release_notes/#version8.1). Evidence was provided by 17,779 ESTs, and 10.5 billion reads across 159 *D. v. virgifera* RNA-seq libraries available in NCBI SRA at time of annotation.

Putative orthologs for Dvir_2.0 protein models were defined against corresponding RefSeq models from *C. elegans* (WBcel_235; GCF_000002985.6), *D. melanogaster* (Release 6; GCF_000001215.4), *A. gambiae* (AgamP3; GCF_000005575.2), *T. castaneum* (Tcas5.2; GCF_000002335.3), and *L decemlineata* (Ldec_2.0; GCF_000500325.1). This was accomplished using OrthoFinder [172, 173]. In brief, sequence similarities were determined by accelerated heuristic searches with the Diamond algorithm [174] to define orthogroups (OGs). Output was used to define orthologous relationships with respect to Dvir_2.0 models. Orthogroups were then used to infer unrooted gene trees DendroBLAST, from which a rooted species tree was generated. Finally, OrthoFinder implemented a duplication-loss-coalescent model to resolve trees and predict gene duplications with lineages.

### Manual annotation and gene family curation

NCBI Dvir_2.0 RefSeq GCF_003013835.1 gene models encoding members of protein families putatively involved in chemical insecticide and Bt pesticidal protein resistance, and olfaction were evaluated further. This involved manual annotation based on available transcript evidence using custom BLAST searches, editing of gene intervals within web Apollo [175] at the i5K workspace (https://i5k.nal.usda.gov/) [176] that were added to RefSeq annotations, and phylogenetic analyses.

Chemosensory genes in the *D. v. virgifera* assembly were annotated through tBLASTn searches using queries of OBP, OR, and IR models from *A.. glabripennis* (Motschulsky) (“Agla”) [177], *L. decemlineata* (Say) (“Ldec”) [100, 113], and *T.castaneum* (Herbst) (“Tcas”) [105]. These initial BLAST searches also included ORs previously annotated from seven other beetle species [73]. A high *E*-value cut-off at 1.0 (ORs/OBPs) and 10.0 (IRs) was used to account for the divergent nature of these genes. Models of corresponding *D. v. virgifera* genes were built manually using Geneious software (Biomatters, Auckland, NZ), and were used in additional BLAST searches until all novel OBP, OR, and IR hits were exhausted. For gene models that were incomplete due to the fractured nature of the assembly, suffixes were added to the gene names according to established protocols (NTE – N-terminus missing; CTE – C-terminus missing; INT – internal sequence missing). A ‘FIX’ suffix was added to gene models where the assembly was repaired manually, and to genes extended using raw reads. ‘JOI’ was added to genes with exons assembled on multiple scaffolds, and ‘PSE’ to putative pseudogenes (i.e., genes with internal stops, frameshifts, missing exons, or missing splice sites). Incomplete genes were usually only promoted to named models if they were > 300 bp (OBPs and ORs) or > 450 bp (IRs). For fragments that did not meet these criteria, sequences were retrieved by BLAST searches with high sequence similarity to ORs and IRs. These orphaned exons likely represent missing portions of existing models and novel genes that could not be annotated in the present effort. In cases where exons of gene models were mis-ordered, interdigitated with other genes, and/or on opposite strands, errors were assumed in most cases to be assembly artifacts and they were reordered correctly within the final model. Nevertheless, these models may be chimeric; such genes are also denoted with the suffix "FIX". In the case of ORs, named models required at least some sequence of the ancestral first exon (“exon A”) [73] that extended through its splice junction with the second exon (“exon B”). These criteria minimized the chance that multiple fragments of the same gene were considered as two separate models.

Multiple sequence alignments were generated among *D. v. virgifera* gene family members and corresponding genes from the related chrysomeliods, *A*. *glabripennis* and *L*. *decemlineata* (excluding short pseudogenes in the case of OBPs and ORs, and all IR pseudogenes) and select conserved genes from *D. melanogaster* [100, 101, 105, 106] (Croset et al. 2010; Vieira and Rozas 2011) using MUSCLE (OBPs and ORs) [178] or MAFFT (IRs) [179]. Any misaligned sequences were adjusted manually, and uninformative regions excised using trimAl v1.2 (http://trimal.cgenomics.org/; settings: similarity threshold 0, gap threshold 0.7, minimum 25% of conserved positions). Trimmed alignments were used to construct phylogenies using FastTree v2.1.10 [180], visualized in FigTree 1.4.4 [181], and formatted using Adobe Illustrator (Adobe Inc., San Jose, CA, USA) and Inkscape 1.0.2–2 (inkscape.org). OBPs, ORs, and divergent IRs were named sequentially based on their position in the phylogeny, but the numbering of divergent IRs started at DvirIR101 to avoid confusion with orthologs in *D*. *melanogaster* (which proceed through IR100a). However, close paralogs situated in tandem arrays were renumbered to follow the apparent order on the scaffold, and antennal IR genes were named according to orthologs in *D*. *melanogaster*, or, in the case of the IR75 group, *L*. *decemlineata*.

TP binding cassette (ABC) transporters are involved in Bt resistance and xenobiotic efflux mechanisms. This gene family was identified by merging evidence from three searches: 1) keyword search of Dvir_2.0 RefSeq protein model fasta headers (gene descriptions) with “ABC transporter” and “multidrug resistance protein”; 2) importation of all *D. v. virgifera* NCBI Dvir_2.0 RefSeq protein models into a local database that was queried with *T. castaneum* orthologs retrieved from the UniProt database (UniProt Consortium 2015; orthologs D2A4C6, D6WES5, D6WK76, D2A4N9, D6WDY5, D6WD20, D2A232, and D6X2Z9) using BLASTp [182], with results filtered for an *E*-value ≤ 10^–20^ and high-scoring segment pairs (HSP) length ≥ 25 amino acids; and 3) query of the above local BLAST database of Dvir_2.0 protein models with 65 ABC transporter protein sequences in silico translated from a prior *D. v virgifera* transcriptome assembly [117] with results filtered at an *E*-value cutoff ≤ 10^–60^ and percent identity ≥ 90%. The unique non-redundant set of putative ABC transporter models with ≤ 80% coverage of corresponding *T. castaneum* orthologs or < 100% coverage of previously predicted *D. v virgifera* ABC transporter proteins were considered putative partial coding regions, and reconstructed in instances when a Dvir_2.0 gene model showed non-overlapping alignment to > 1 query. For these partial coding regions, scaffold positions of putative ABC transporter models were retrieved from the RefSeq gff3 file (GCF_003013825.1_Dvir_v2.0_protein.faa). Specifically, fragments of a given *D. v. virgifera* transcript alignment or *T. castaneum* ortholog located across ≥ 2 scaffolds were denoted “Part X of Y” in comments (X is order of fragment among the total of Y predicted fragments comprising the predicted full CDS).

Phylogenetic relationships among putative *D. v virgifera* ABC transporters and a representative *T. castaneum* orthologs in each subfamily (A–H) were constructed using MEGA8.0 [183] from a multiple amino acid sequence alignment of the translated nucleotide binding domain (NBD) using the ClustalW algorithm [184] using default parameters. The MEGA8.0 “Best Model” utility [183] was used to determine the optimal LG substitution model [185] with gamma-distributed rates (“LG + G + I”) that maximized the Bayesian Information Criterion (BIC) score when an empirically-derived gamma distribution was applied and alignment gaps were ignored. A subsequent Maximum-Likelihood (ML) approach was used to reconstruct an unrooted phylogeny with the consensus tree constructed from 1,000 bootstrap pseudo-replicates of the aligned NBD. The resulting consensus phylogeny was output in Newick (.nwk) format, and annotated using Interactive Tree of Life (iTOL) v5 [186] (https://itol.embl.de/).

Cytochrome P450 (*cyp*) gene family members are involved in a wide range of biochemical functions including xenobiotic detoxification. Putative P450s within *D. v. virgifera* RefSeq GCF_003013835.1 protein models were identified by BLAST searches of the complete set of *D. v. virgifera* proteins using known cytochrome P450 sequences from *T. castaneum, L. decemlineata* and *A. glabripennis* as queries. A nonredundant list of hits from *Diabrotica* was compiled for manual analysis. Each putative P450 was submitted as a query in a BLAST search against the Arthropoda database in UniProt KB [187] and an InterProScan search [188] to confirm or discount their identity as legitimate cytochrome P450. Gene models were deemed complete if they predicted a protein of a size typical of cytochrome P450s (400 – 550 amino acid residues), contained the characteristic N-terminal transmembrane domain, and heme-binding domain and were terminated by a valid stop codon. Cases in which the RefSeq gene model consisted of two adjacent genes fused together were identified as abnormally large predicted proteins with duplicates of the transmembrane and/or heme-binding domains.

Verified complete cytochrome P450s were placed into CYP families based on amino acid identity with previously defined members [189]. Complete cytochrome P450s were aligned using the motif-aware aligner PRALINE [190–192] at the PRALINE web server (https://www.ibi.vu.nl/programs/pralinewww/) [191] (Parameters: BLOSUM62 exchange weights matrix, gap opening penalty = 15, gap extension penalty = 1. PSI-BLAST pre-profile processing using the NCBI nr database with 3 iterations and *E*-value cutoff ≤ 0.01). Putative secondary structures were defined by comparison to the Dictionary of Secondary Structure of Proteins (DSSP) database [193] using the PSI-blast based secondary structure PREDiction (PSIPRED) algorithm with the PSIPRED Server (http://bioinf.cs.ucl.ac.uk/psipred/) [194]. Putative transmembrane regions were predicted using a hidden Markov model in TMHMM 2.0 [195] with default parameters. The resulting alignment was loaded into the R statistical environment [196], and a matrix of pairwise amino acid identities calculated using the seqinr package [197]. Aligned sequences with ≥ 40% amino acid identity between members were clustered using the base R function hclust with the single-linkage method. These clusters were then assigned to CYP families by query of representative *D. v. virgifera* cluster members against the Arthropoda section of the UniProt KB database [187] via the UniProt web server (https://www.uniprot.org/blast/). Clusters were assigned to a CYP family if the query hit had ≥ 40% identity to a full-length *T. castaneum*, *L. decemlineata*, or *D. ponderosae* CYP protein in the “reviewed” section of UniProt KB.

An unrooted ML-based phylogeny was reconstruction for all putative full-length *D. v. virgifera* CYP protein sequences and representative arthropod CYP family members. *Diabrotica* and representative CYP sequences were first aligned using PRALINE as described above and imported into MEGA [183] to determine the optimal substitution model as the LG model [185] with gamma-distributed rates (“LG + G + I + F”). Node support for the subsequent ML-based phylogeny was based on 500 bootstrap pseudoreplicates, with the consensus tree topology output in newick (.nwk) format and annotated using iTOL v5 [186] (https://itol.embl.de/).

### Differential gene expression in response to larval host plant feeding

Seeds for open-pollinated yellow dent maize (Hancock Farm and Seed Co., Inc, Dale City, FL, USA), and *S. bicolor* (BCK60) and *P. virgatum* (KxS) varieties (provided by Dr. Tiffany Heng-Moss, University of Nebraska, Lincoln, NE, USA) were germinated in standard potting soil and grown in a growth chamber maintained at a constant temperature of 21 ºC and a 12-h photoperiod. *Miscanthus* (*Miscanthus* x *giganteus*) rhizomes (Maple River Farms, Owosso, MI, Michigan) were grown under identical conditions. Eggs from the non-diapausing *D. v. virgifera* laboratory strain [154] maintained at USDA-ARS, NCARL, Brookings, SD, USA, were incubated in soil at 25 ºC for 14 days. Eggs were then cleaned and surface sterilized by washing for 3 min in 40 ml Lysol multi-purpose cleaner (Reckitt Benckiser, Inc., Parsippany, NJ, USA), 2 min in 10% formaldehyde, 1.5 min in 1% bleach (Clorox, Oakland, CA, USA), and then rinsed twice with distilled water. Cleaned eggs were placed on filter paper moistened with distilled water in Petri dishes and incubated at 23 ºC until hatch. Neonates (< 12 h post-hatch) were transferred to a Petri dish containing freshly extracted host plant roots (maize, *Miscanthus*, *P. virgatum*, or *S. bicolor*) or starved for either 6 or 12 h. After the designated time points, neonates observed feeding on host roots were collected (*n* = 15 individuals per sample across 4 replicates per host or starvation exposure; 5 treatments × 4 replicates × 2 exposure times; 40 total), flash frozen in liquid nitrogen, and stored at -80 ºC.

Total RNA was isolated from each sample (*n* = 40) using the RNeasy Mini Kit according to the manufacturer’s instructions (Qiagen, Hilden, Germany), and quality checked using at Bioanalyzer System 2100 (Agilent, Santa Clara, CA, USA). Libraries were prepared using the TruSeq RNA Library Prep Kit v2 (Illumina) and 100-base single-end reads generated on an Illumina HiSeq2000 Sequencing System at the University of Nebraska Medical Centre (Omaha, NE, USA). Quality of subsequence sequence reads was checked using FastQC v0.11.5 [198]. Reads were checked for per base sequence quality, per base sequence quality scores, per base sequence content, per sequence guanine and cytosine (GC) content, sequence length, and sequence distribution.

Estimated abundance of reads assigned to a transcript were then quantified across all gene model derived transcripts using Kallisto v 0.46.1 [199]. For this, the transcripts for the *D. v. virgifera* genome were downloaded from NCBI (Accession: GCF_003013835.1) in FASTA format and processed with the Kallisto "index" function (k = 31). Reads from each of the 40 *D. v. virgifera* libraries were pseudo aligned to transcripts and quantified using default parameters in the Kallisto "quant" function for single-end reads (fragment length = 200 and sd = 20). Kallisto was then used to construct 100 bootstrap pseudoreplicates to estimate technical variance and evaluate the probability of correct read assignments to the transcripts.

Significance of any differences among abundance of reads between bootstrap samples generated by Kallisto for each treatment were assessed using the Sleuth package v 0.30 [200] in R 4.02 [196]. Differential transcript abundances were estimated using the likelihood ratio test (LRT) and the Wald test [201] using default parameters. The Wald tests were performed on all pairwise combinations of treatments (maize), alternative host (*Miscanthus*), marginal host (*P. virgatum*), poor host (*S. bicolor*) and starvation at both 6- and 12-h exposure timepoints. Thresholds for statistical significance were set at an α ≤ 0.05. Differentially-expressed transcripts were reported as beta values equivalent to log2 fold-change, accounting for technical variability of transcripts [200]. Principal component analysis (PCA) of gene expression data was performed using functions provided by the Sleuth package.

Putative functional annotations were assigned to corresponding protein models for differentially-expressed transcripts using GOAnna [202] using default settings with the invertebrate subsection of the UniProt database. The gene ontologies (GOs) in the sliminput.txt files generated by GOanna were used as input for GOSlimViewer to parse and summarize molecular function (F), biological process (P), and cellular component (C) at level 2. Annotations were also converted to gene annotation format (.gaf) file using Goanna2ga. Gene Ontology (GO) enrichment analyses were performed on the differentially-expressed genes using the R Bioconductor package goseq [203], which takes length bias into account when performing analysis [57]. For this enrichment analysis, a gene association file was produced indicating the transcript length extracted from the feature table (Accession: GCF_003013835.1) and the GO term(s) in the.gaf file generated above using the GOanna pipeline. GO term enrichment analyses were performed individually on both the up- and down-regulated transcripts. GO terms were declared significantly over-represented at a Benjamini and Hochberg (BH) determined false discovery rate (FDR) ≤ 0.1 [204].

## Supplementary Information

**Additional file 1:** **Supplementary Table S1.** Genomic libraries and sequencing reads used in *Diabrotica virgifera virifera* genome assembly of samples from inbred strain Ped12 (BioProject PRJNA432972; BioSample SAMN08631342). Count in millions of paired end (PE) reads.

**Additional file 2:** **Supplementary Figure S1.** Analysis of the 31-nucleotide k-mer distribution among short paired-end reads from 0.5 kb *Diabrotica virgifera virgifera* genomic insert libraries (SRA accessions: SRR6985753 to SRR6985756). Distributions shown for **A)** linear and **B)** log plots. These provided minimum (min) and maximum (max) estimates for genome haploid (1N) length and proportions comprising unique and repeated sequences. Estimated mean heterozygosity and duplication level was 0.448% and 0.331%, respectively. Low coverage area <10 under the red curve indicative of sequence error.

**Additional file 3: Supplementary Table S2.** Joins made between scaffolds from Dvir_1.0 by L-RNA-Scaffolder based on assembled transcripts in the National Center for Biotechnology Information Transciptome Shotgun Assemby accession GHNJ00000000.1.

**Additional file 4: Supplementary Table S3.** Joins made between scaffolds from Dvir_1.0 by L-RNA-Scaffolder based on assembled transcripts in the National Center for Biotechnology Information Transciptome Shotgun Assemby accession ERZ1775117.1.

**Additional file 5:** **Supplementary Table S4.** Comparison of the *Diabrotica virgifera virgifera* reference genome assembly, Dvir_2.0, to assemblies from other coleopteran species in the Family Chrysomelidae. NA = not available; scaffolding not performed.

**Additional file 6:** **Supplementary Figure S2.** Assessments of coverage, mapping and scaffolding results for reads generated from Dovetail Chicago® libraries (**supplementary Table S1**). **A)** Calculated distribution of coverage depth among library reads, with an overall combined mean of ~12.11-fold. **B)** Comparison of the distribution among scaffold sizes between Dvir_1.0 (SOAPdenovo + L_RNA_Scaffolder) and the scaffolded Dvir_2.0 assembly. **C)** Estimated distribution of insert sizes for three Chicago® libraries.

**Additional file 7. Supplementary Table S5a.** Orthologous groups predicted by Orthofinder2. Output from orthogroups.tsv.

**Additional file 8: Supplementary Table S5b.** Shared orthologous groups predicted by Orthofinder2. Input for Venn diagram in Figure 2B.

**Additional file 9: Supplementary Table S6.** Manually annotated chemosensory protein genes in the Dvir_2.0 assembly. Complete and fragmented models for A) B) odorant binding protein (OBP), C) D) odorant receptor (OR), and E) F) ionotropic receptor (IR) gene family members.

**Additional file 10: Supplementary Table S7.** Manually annotated genes encoding ATP binding cassette (ABC) transporter proteins in the Dvir_2.0 assembly. Data shown for A) Translations from *Diabrotica virgifera virgifera* Dvir_v2.0 RefSeq GCF_003013835.1 v100 gene models, B) BLAST results for previously annotated *D. v. virgifera *ABC transporters against Dvir_v2.0 RefSeq GCF_003013835.1 v100 gene models, and C) putative *D. v. virgifera* ABC transporter subfamily assignments.

**Additional file 11: Supplementary Table S8.** Putative cytochrome P450s described from among Dvir_v2.0 RefSeq GCF_003013835.1 v100 gene models. A) 88 putative full-length gene models, and B) putative *D. v. virgifera* cytochrome P450s subfamily assignments.

**Additional file 12: Supplementary Table S9.** RNA sequencing (RNA-seq) reads generated from 4 replicates of pooled *Diabrotica virgifera virgifera* larvae across 8 different plant exposure by duration treatments (*n* = 40). Single end (SE) read data accessioned under NCBI SRA experiment SRP131734 and BioProject PRJNA429767.

**Additional file 13: Supplementary Table S10.** Predicted differences in Dvir_v2.0 RefSeq GCF_003013835.1 v100 transcript model abundances after 12-hour exposures of *Diabrotica virgifera virgifera* larvae on maize compared to A) *Miscanthus*, B) *Sorghum bicolor*, C) *Panicum virgatum* and D) starvation conditions. Results shown for predicted p-value, and B-H adjusted p-value, and fold-change.

**Additional file 14: Supplementary Table S11.** Gene ontology (GO) terms assigned to transcripts predicted to be significantly A) up-regulated and B) down-regulated.

## Data Availability

The sequence reads used for assembly and scaffolding of the *D. v. virgifera* genome (National Center for Biotechnology Information (NCBI) BioProject PRJNA432972) are in NCBI Sequence Read Archive (SRA) accessions SRX1428743-SRX1428749 and SRX3924093-SRX3924098, and 14,289,743–14,289,745, respectively. The assembled Dvir_2.0 contig and scaffolded sequences are available in the GenBank accessions PXJM00000000.2 (contigs: PXJM02000001-PXJM02585680; scaffolds ML014983-ML058324), where the latter comprise the GenBank assembly accession GCA_003013835.2. Gene ontologies for *D. v. virgifera* RefSeq protein models generated via GOanna are available at the Ag Data Commons (https://data.nal.usda.gov/) under https://doi.org/10.15482/USDA.ADC/1524777. RNA-seq read data applied for estimates of differential expression are available in SRA accessions SRX3628070 to SRX3628109.

## Abbreviations

ABC: ATP binding cassette
BP: Biological process
Bt: *Bacillus thuringiensis*
BUSCO: Benchmarking Universal Single-Copy Orthologs
CC: Cellular component
CYP: Cytochrome P450
DEG: Differentially-expressed gene
GO: Gene ontology
IR: Ionotropic receptor
MF: Molecular function
MP: Mate pair
OBP: Odorant binding protein
OGs: Orthogroups
OR: Odorant receptor
PE: Paired end
RNA-seq: RNA sequence
