# Supplementary material for: A draft Diabrotica virgifera virgifera genome: insights into control and host plant adaption by a major maize pest insect
Source: BMC Genomics. 2023 Jan 13;24:19. doi: 10.1186/s12864-022-08990-y (PMC9840275; doi:10.1186/s12864-022-08990-y)
Supplement: Supplementary file 1 — Additional file 1: Supplementary Table S1. Genomic libraries and sequencing reads used in Diabrotica virgifera virifera genome assembly of samples from inbred strain Ped12 (BioProject PRJNA432972; BioSample SAMN08631342). Count in millions of paired end (PE) reads. [file 12864_2022_8990_MOESM1_ESM.docx]

**Supplementary Table S1** Genomic libraries and sequencing reads used in *Diabrotica virgifera virgifera* genome assembly of samples from inbred strain Ped12 (BioProject PRJNA432972; BioSample SAMN08631342). Count in millions of paired end (PE) reads.

|  |  |  | Insert | HiSeq^#^ |  | Read | |  | Raw reads | |
| --- | --- | --- | --- | --- | --- | --- | --- | --- | --- | --- |
| Source(s) | Library name | Library Type | (kb) | Lanes |  | Type | Len |  | Count/Gb | SRA accession/SRA run |
| ♀ #1 | Diabrotica virgifera virgifera_500bp | Shotgun | 0.5 | 9^#^ |  | PE | 100 |  | 149.3/29.9 | SRX3924098/SRR6984002 |
|  |  | paired-end |  |  |  |  |  |  | 183.7/36.7 | SRX3924098/SRR6985753 |
|  |  |  |  |  |  |  |  |  | 138.5/27.7 | SRX3924098/SRR6985754 |
|  |  |  |  |  |  |  |  |  | 116.6/23.3 | SRX3924098/SRR6985755 |
|  |  |  |  |  |  |  |  |  | 373.4/74.7 | SRX3924098/SRR6985756 |
|  |  |  |  |  |  |  |  |  | 167.5/24.9 | SRX3924098/SRR7011719 |
|  |  |  |  |  |  |  |  |  | 168.2/18.9 | SRX3924098/SRR7011720 |
|  |  |  |  |  |  |  |  |  | 176.4/16.0 | SRX3924098/SRR7011721 |
|  |  |  |  |  |  |  |  |  | 176.4/45.6 | SRX3924098/SRR7011722 |
| ♀ #1 | Diabrotica virgifera virgifera_1.5kb | Mate pair | 1.5 | 1^#^ |  | PE | 100 |  | 119.0/23.8 | SRX3924097/SRR6984003 |
| ♀ #2 & #3 | Diabrotica virgifera virgifera_5kb | Mate pair | 5.0 | 1^#^ |  | PE | 100 |  | 189.8/38.0 | SRX3924096/SRR6984004 |
| ♀ #2 & #3 | Diabrotica virgifera virgifera_10kb | Mate pair | 10.0 | 2^#^ |  | PE | 100 |  | 43.9/ 8.8 | SRX3924095/SRR6984005 |
|  |  |  |  |  |  |  |  |  | 64.4/12.8 | SRX3924095/SRR6985758 |
| ♀ #4 & #5 | Diabrotica virgifera virgifera_15kb | Mate pair | 15.0 | 1^#^ |  | PE | 100 |  | 167.3/33.5 | SRX3924094/SRR6984006 |
| ♀ #6 & #7 | Dovetail Chicago® library 001 | Chicago® paired-end | 100.0 | 3* |  | PE | 150 |  | 142.6/42.8 | SRX14289743/SRR18141732 |
|  | Dovetail Chicago® library 002 | Chicago® paired-end |  |  |  |  |  |  | 157.2/47.2 | SRX14289744/SRR18141731 |
|  | Dovetail Chicago® library 003 | Chicago® paired-end |  |  |  |  |  |  | 135.5/40.6 | SRX14289745/SRR18141730 |

# Illumina HiSeq2000; * Illumina HiSeqX
