# Supplementary material for: A draft Diabrotica virgifera virgifera genome: insights into control and host plant adaption by a major maize pest insect
Source: BMC Genomics. 2023 Jan 13;24:19. doi: 10.1186/s12864-022-08990-y (PMC9840275; doi:10.1186/s12864-022-08990-y)
Supplement: Supplementary file 2 — Additional file 2: Supplementary Figure S1. Analysis of the 31-nucleotide k-mer distribution among short paired-end reads from 0.5 kb Diabrotica virgifera virgifera genomic insert libraries (SRA accessions: SRR6985753 to SRR6985756). Distributions shown for A) linear and B) log plots. These provided minimum (min) and maximum (max) estimates for genome haploid (1N) length and proportions comprising unique and repeated sequences. Estimated mean heterozygosity and duplication level was 0.448% and 0.331%, respectively. Low coverage area <10 under the red curve indicative of sequence error. [file 12864_2022_8990_MOESM2_ESM.docx]

**Supplementary Figure S1** Analysis of the 31-nucleotide k-mer distribution among short paired-end reads from 0.5 kb *Diabrotica virgifera virgifera* genomic insert libraries (SRA accessions: SRR6985753 to SRR6985756). Distributions shown for **A)** linear and **B)** log plots. These provided minimum (min) and maximum (max) estimates for genome haploid (1N) length and proportions comprising unique and repeated sequences. Estimated mean heterozygosity and duplication level was 0.448% and 0.331%, respectively. Low coverage area <10 under the red curve indicative of sequence error.

**
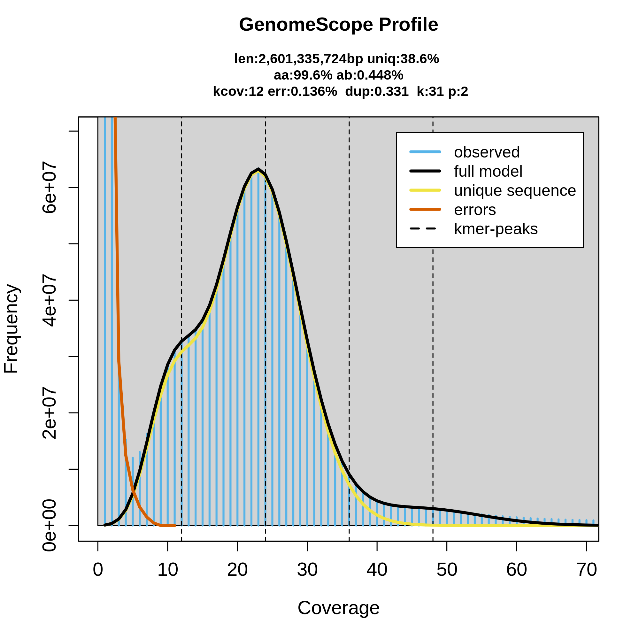
**
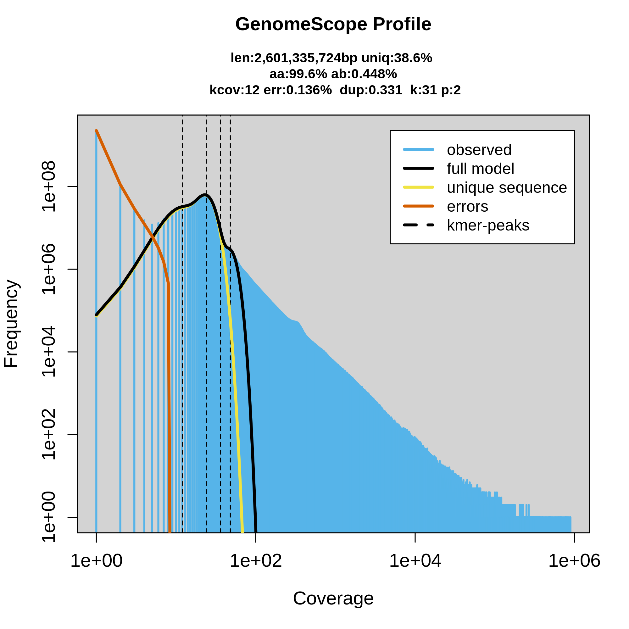


| property | min | max |
| --- | --- | --- |
| Homozygous (aa) | 99.5357% | 99.5686% |
| Heterozygous (ab) | 0.431413% | 0.46431% |
| Genome Haploid Length | 2,589,652,882 bp | 2,601,335,724 bp |
| Genome Repeat Length | 1,590,100,175 bp | 1,597,273,681 bp |
| Genome Unique Length | 999,552,707 bp | 1,004,062,043 bp |
| Model Fit | 43.1855% | 99.035% |
| Read Error Rate | 0.135869% | 0.135869% |
