# Supplementary material for: A draft Diabrotica virgifera virgifera genome: insights into control and host plant adaption by a major maize pest insect
Source: BMC Genomics. 2023 Jan 13;24:19. doi: 10.1186/s12864-022-08990-y (PMC9840275; doi:10.1186/s12864-022-08990-y)
Supplement: Supplementary file 5 — Additional file 5: Supplementary Table S4. Comparison of the Diabrotica virgifera virgifera reference genome assembly, Dvir_2.0, to assemblies from other coleopteran species in the Family Chrysomelidae. NA = not available; scaffolding not performed. [file 12864_2022_8990_MOESM5_ESM.docx]

**Supplementary Table S4** Comparison of the *Diabrotica virgifera virgifera* reference genome assembly, Dvir_2.0, to assemblies from other coleopteran species in the Family Chrysomelidae. NA = not available; scaffolding not performed.

| Metric | | Dvir_1.0 | Dvir_2.0^1^ | Ldec_2.0^2^ | icDCau_1.0^3^ | ULB_GQUI_1.0^4^ | Cmac^5^ | Ocom^6^ | AV1.0^7^ |
| --- | --- | --- | --- | --- | --- | --- | --- | --- | --- |
| Contig assembly | |  |  |  |  |  |  |  |  |
|  | Total number | 582,895 | 586,720 | 45,556 | 7,323 | 10,033 | 15,778 | 7,003 | 17,580 |
|  | Length (bp) | 1,852,749,179 | 1,850,691,159 | 633,896,204 | 315,178,479 | 1,731,947,787 | 1,007,816,681 | 774,411,302 | 844,331,391 |
|  | N50 (Mb) | 6,286 | 6,227 | 46,596 | 62,896 | 432,124 | 212,245 | 195,463 | 107,784 |
|  | L50 | 83,830 | 84,395 | 3,600 | 1,431 | 1,130 | 1,183 | 925 | 2,104 |
|  | N90 (bp) | 1,602 | 1,581 | 3,324 | 20,599 | 81,292 | 26,507 | 42,523 | 26,046 |
|  | L90 | 310,265 | 313,452 | 35,359 | 4,955 | 4,593 | 6,491 | 4,614 | 8,009 |
|  | Largest contig (bp) | 79,911 | 79,911 | 439,106 | 575,256 | 3,029,505 | 2,070,929 | 5,110,064 | 1,698,099 |
| Scaffold assembly | |  |  |  |  |  |  |  |  |
|  | Total number | 164,564 | 87,712 | 26,908 | 1,191 | NA | NA | NA | 4,479 |
|  | Length (bp) | 2,412,004,911 | 2,418,073,815 | 641,992,784 | 315,349,852 | NA | NA | NA | 864,762,032 |
|  | Gaps | 418,331 | 499,008 | 54,451 | 6,213 | NA | NA | NA | 11,830 |
|  | GC content (%) | 36.5 | 36.5 | 35.02 | 31.89 | NA | NA | NA | 30.92 |
|  | N bases | 559,255,732 | 567,382,656 | 8,096,580 | 171,373 | NA | NA | NA | 20,430,641 |
|  | N50 (bp) | 95,776 | 489,108 | 139,046 | 805,047 | NA | NA | NA | 557,265 |
|  | L50 | 5,977 | 1,128 | 1,179 | 97 | NA | NA | NA | 429 |
|  | N90 (bp) | 5,258 | 37,112 | 23,706 | 109,963 | NA | NA | NA | 122,770 |
|  | L90 | 42,737 | 7,527 | 5,389 | 512 | NA | NA | NA | 1,669 |
|  | Largest (bp) | 1,176,379 | 8,068,850 | 1,851,299 | 6,246,341 | NA | NA | NA | 6,674,939 |
|  | BUSCOs (arthropoda_odb10) |  |  |  |  |  |  |  |  |
|  | Complete (C) | 934 (92.2%) | 948 (93.6%) | 953 (94.1%) | 945 (93.3%) | 988 (97.6%) | 911 (89.9%) | 984 (97.2%) | 1000(98.7%) |
|  | Complete single copy (S) | 917 (90.5%) | 931 (91.9%) | 937 (92.5%) | 938 (92.6%) | 967 (95.5%) | 862 (85.1%) | 877 (86.6%) | 929 (91.7%) |
|  | Complete duplicated (D) | 17 ( 1.7%) | 17 ( 1.7%) | 16 ( 1.6%) | 7 ( 0.7%) | 21 ( 2.1%) | 49 ( 4.8%) | 107 (10.6%) | 71 ( 7.0%) |
|  | Fragmented (F) | 60 ( 5.9%) | 46 ( 4.5%) | 35 ( 3.5%) | 15 ( 1.5%) | 12 ( 1.2%) | 43 ( 4.2%) | 5 ( 0.5%) | 3 ( 0.3%) |
|  | Missing (M) | 19 ( 1.9%) | 19 ( 1.9%) | 25 (2.4%) | 53 ( 5.2%) | 13 ( 1.2%( | 59 ( 5.9%) | 24 ( 2.3%) | 10 ( 1.0%) |

1. *Diabrotica virgifera virgifera* WGS Project: PXJM02; GenBank assembly accession: GCA_003013835.2 *(assembly described herin)*
2. *Leptinotarsa decemlineata*; WGS Project: AYNB02; GenBank assembly accession: GCA_000500325.2 (Schoville et al. 2018)
3. *Diorhabda carinulata*; WGS Project: JAJJBS01; GenBank assembly accession: GCA_020975425.1 (University of Idaho, unpublished)
4. *Gonioctena quinquepunctata*; WGS Project: JAFIRS01; GenBank assembly accession: GCA_018342105.1 (Lukicheva et al. 2021)
5. *Callosobruchus maculatus*; WGS Project: CAACVG01; GenBank assembly accession: GCA_900659725.1 (Sayadi et al., 2019)
6. *Ophraella communa*; WGS Project: CACRSI01; GenBank assembly accession: GCA_902651945.1 (Bouchemousse et al. 2020)
7. *Altica viridicyanea:* National Genomics Data Center accession number: GWHAMMQ00000000 (Xue et al. 2021)
