# Supplementary material for: A draft Diabrotica virgifera virgifera genome: insights into control and host plant adaption by a major maize pest insect
Source: BMC Genomics. 2023 Jan 13;24:19. doi: 10.1186/s12864-022-08990-y (PMC9840275; doi:10.1186/s12864-022-08990-y)

**Supplementary Figure S2** Assessments of coverage, mapping and scaffolding results for reads generated from Dovetail Chicago® libraries (**supplementary Table S1**). **A)** Calculated distribution of coverage depth among library reads, with an overall combined mean of ~12.11-fold. **B)** Comparison of the distribution among scaffold sizes between Dvir_1.0 (SOAPdenovo + L_RNA_Scaffolder) and the scaffolded Dvir_2.0 assembly. **C)** Estimated distribution of insert sizes for three Chicago® libraries.


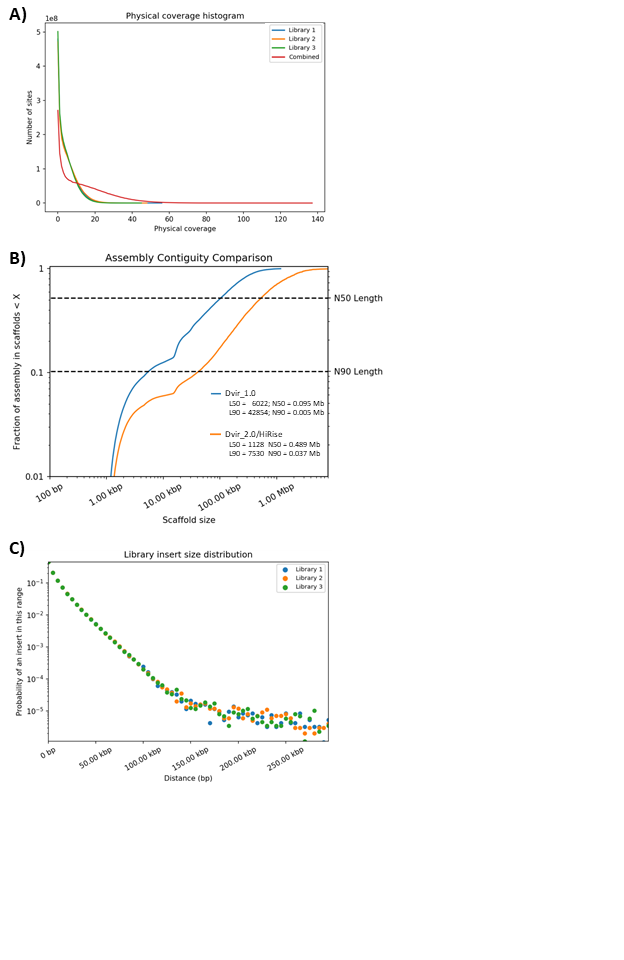

Supplement: Supplementary file 6 — Additional file 6: Supplementary Figure S2. Assessments of coverage, mapping and scaffolding results for reads generated from Dovetail Chicago® libraries (supplementary Table S1). A) Calculated distribution of coverage depth among library reads, with an overall combined mean of ~12.11-fold. B) Comparison of the distribution among scaffold sizes between Dvir_1.0 (SOAPdenovo + L_RNA_Scaffolder) and the scaffolded Dvir_2.0 assembly. C) Estimated distribution of insert sizes for three Chicago® libraries. [file 12864_2022_8990_MOESM6_ESM.docx]
