# Supplementary material for: A draft Diabrotica virgifera virgifera genome: insights into control and host plant adaption by a major maize pest insect
Source: BMC Genomics. 2023 Jan 13;24:19. doi: 10.1186/s12864-022-08990-y (PMC9840275; doi:10.1186/s12864-022-08990-y)
Supplement: Supplementary file 12 — Additional file 12: Supplementary Table S9. RNA sequencing (RNA-seq) reads generated from 4 replicates of pooled Diabrotica virgifera virgifera larvae across 8 different plant exposure by duration treatments (n = 40). Single end (SE) read data accessioned under NCBI SRA experiment SRP131734 and BioProject PRJNA429767. [file 12864_2022_8990_MOESM12_ESM.docx]

**Supplementary Table S6** RNA sequencing (RNA-seq) reads generated from 4 replicates of pooled *Diabrotica virgifera virgifera* larvae across 8 different plant exposure by duration treatments (*n* = 40). Single end (SE) read data accessioned under NCBI SRA experiment SRP131734 and BioProject PRJNA429767.

|  |  |  |  |  | Read | |  | Raw reads | |
| --- | --- | --- | --- | --- | --- | --- | --- | --- | --- |
| Treatment Time (hr) | | Library | BioSample |  | Type Len (bp) | |  | Count/Gb | SRA accession/SRA run |
| Maize | 6 | WCR_WF | SAMN08358866 |  | SE | 100 |  | 1.27/0.722 | SRX3628101/SRR6650448 |
| Maize | 6 | WCR_WB | SAMN08358850 |  | SE | 100 |  | 1.32/0.755 | SRX3628097/SRR6650452 |
| Maize | 6 | WCR_WI | SAMN08358881 |  | SE | 100 |  | 1.21/0.683 | SRX3628087/SRR6650462 |
| Maize | 6 | WCR_WA | SAMN08358627 |  | SE | 100 |  | 1.38/0.779 | SRX3628072/SRR6650477 |
| *Miscanthus giganteus* | 6 | WCR_WM | SAMN08358890 |  | SE | 100 |  | 1.03/0.574 | SRX3628091/SRR6650458 |
| *Miscanthus giganteus* | 6 | WCR_WK | SAMN08358885 |  | SE | 100 |  | 1.12/0.623 | SRX3628089/SRR6650460 |
| *Miscanthus giganteus* | 6 | WCR_WL | SAMN08358888 |  | SE | 100 |  | 0.92/0.517 | SRX3628088/SRR6650461 |
| *Miscanthus giganteus* | 6 | WCR_WJ | SAMN08358883 |  | SE | 100 |  | 1.26/0.709 | SRX3628086/SRR6650463 |
| *Sorghum bicolor* | 6 | WCR_WD | SAMN08358863 |  | SE | 100 |  | 1.31/0.741 | SRX3628099/SRR6650450 |
| *Sorghum bicolor* | 6 | WCR_WC | SAMN08358851 |  | SE | 100 |  | 1.37/0.773 | SRX3628098/SRR6650451 |
| *Sorghum bicolor* | 6 | WCR_WN | SAMN08358891 |  | SE | 100 |  | 1.42/0.790 | SRX3628090/SRR6650459 |
| *Sorghum bicolor* | 6 | WCR_WA2 | SAMN08358661 |  | SE | 100 |  | 1.40/0.803 | SRX3628075/SRR6650474 |
| Starvation | 6 | WCR_WE | SAMN08358865 |  | SE | 100 |  | 1.42/0.800 | SRX3628100/SRR6650449 |
| Starvation | 6 | WCR_WG | SAMN08358874 |  | SE | 100 |  | 1.42/0.804 | SRX3628085/SRR6650464 |
| Starvation | 6 | WCR_WH | SAMN08358876 |  | SE | 100 |  | 1.21/0.681 | SRX3628084/SRR6650465 |
| Starvation | 6 | WCR_WO | SAMN08358894 |  | SE | 100 |  | 1.35/0.754 | SRX3628081/SRR6650468 |
| *Panicum virgatum* | 6 | WCR_WA9 | SAMN08358848 |  | SE | 100 |  | 1.45/0.829 | SRX3628096/SRR6650453 |
| *Panicum virgatum* | 6 | WCR_WA8 | SAMN08358847 |  | SE | 100 |  | 1.37/0.790 | SRX3628095/SRR6650454 |
| *Panicum virgatum* | 6 | WCR_WA13 | SAMN08358659 |  | SE | 100 |  | 1.22/0.697 | SRX3628077/SRR6650472 |
| *Panicum virgatum* | 6 | WCR_WA14 | SAMN08358660 |  | SE | 100 |  | 1.32/0.755 | SRX3628074/SRR6650475 |
| Maize | 12 | WCR_WR | SAMN08358897 |  | SE | 100 |  | 1.32/0.735 | SRX3628103/SRR6650446 |
| Maize | 12 | WCR_WQ | SAMN08358896 |  | SE | 100 |  | 1.28/0.715 | SRX3628102/SRR6650447 |
| Maize | 12 | WCR_WS | SAMN08358922 |  | SE | 100 |  | 1.39/0.778 | SRX3628082/SRR6650467 |
| Maize | 12 | WCR_WP | SAMN08358893 |  | SE | 100 |  | 1.24/0.695 | SRX3628080/SRR6650469 |
| *Miscanthus giganteus* | 12 | WCR_WY | SAMN08359041 |  | SE | 100 |  | 1.71/0.976 | SRX3628108/SRR6650441 |
| *Miscanthus giganteus* | 12 | WCR_WX | SAMN08358991 |  | SE | 100 |  | 1.35/0.773 | SRX3628105/SRR6650444 |
| *Miscanthus giganteus* | 12 | WCR_WW | SAMN08358950 |  | SE | 100 |  | 1.26/0.722 | SRX3628104/SRR6650445 |
| *Miscanthus giganteus* | 12 | WCR_WT | SAMN08358923 |  | SE | 100 |  | 1.34/0.748 | SRX3628083/SRR6650466 |
| *Sorghum bicolor* | 12 | WCR_WV | SAMN08358926 |  | SE | 100 |  | 1.23/0.714 | SRX3628107/SRR6650442 |
| *Sorghum bicolor* | 12 | WCR_WU | SAMN08358924 |  | SE | 100 |  | 1.18/0.680 | SRX3628106/SRR6650443 |
| *Sorghum bicolor* | 12 | WCR_WA12 | SAMN08358631 |  | SE | 100 |  | 1.42/0.815 | SRX3628076/SRR6650473 |
| *Sorghum bicolor* | 12 | WCR_WA11 | SAMN08358630 |  | SE | 100 |  | 1.41/0.805 | SRX3628071/SRR6650478 |
| Starvation | 12 | WCR_WZ | SAMN08359095 |  | SE | 100 |  | 1.27/0.735 | SRX3628109/SRR6650440 |
| Starvation | 12 | WCR_WA7 | SAMN08358846 |  | SE | 100 |  | 1.35/0.774 | SRX3628094/SRR6650455 |
| Starvation | 12 | WCR_WA1 | SAMN08358628 |  | SE | 100 |  | 1.20/0.691 | SRX3628073/SRR6650476 |
| Starvation | 12 | WCR_WA10 | SAMN08358629 |  | SE | 100 |  | 1.51/0.868 | SRX3628070/SRR6650479 |
| *Panicum virgatum* | 12 | WCR_WA6 | SAMN08358845 |  | SE | 100 |  | 1.22/0.698 | SRX3628093/SRR6650456 |
| *Panicum virgatum* | 12 | WCR_WA5 | SAMN08358843 |  | SE | 100 |  | 1.48/0.849 | SRX3628092/SRR6650457 |
| *Panicum virgatum* | 12 | WCR_WA4 | SAMN08358842 |  | SE | 100 |  | 1.55/0.888 | SRX3628079/SRR6650470 |
| *Panicum virgatum* | 12 | WCR_WA3 | SAMN08358663 |  | SE | 100 |  | 1.38/0.794 | SRX3628078/SRR6650471 |
